# The Sol–Gel Metal-Oxide Skeleton Affects the Catalytic Properties of In Situ Formed Metal Nanoparticles

**DOI:** 10.1021/acsomega.5c07438

**Published:** 2026-02-17

**Authors:** Kavya Vidyadharan, Dan Meyerstein, Ariela Burg, Amir Mizrahi, Jennifer Strunk, Yael Albo

**Affiliations:** † Chemical Sciences Department and the Radical Research Center, 42732 Ariel University, Ariel 4070000, Israel; ‡ Chemistry Department, 26732 Ben-Gurion University, Beer-Sheva 8410501, Israel; § Chemical Engineering Department, 42740 Sami Shamoon College of Engineering, Beer-Sheva 84100, Israel; ∥ Nuclear Research Centre Negev, Beer-Sheva 84190, Israel; ⊥ Industrielle Chemie und Heterogene Katalyse, Technische Universität München, Lichtenbergstr. 4, Garching bei München 85748, Germany; # Chemical Engineering Department and the Radical Research Center, Ariel University, Ariel 4070000, Israel

## Abstract

The interaction between the mesoporous metal-oxide matrix and the adsorbed/entrapped active metal species attracts attention and curiosity due to its critical role in defining catalytic performance. Herein, the effects of TiO_2_, Al_2_O_3_, and SiO_2_ sol–gel matrices on the catalytic properties of encapsulated Cu^0^ and Ni^0^ species catalyzing the dehalogenation of the hazardous disinfection byproducts, chloro and bromoacetic acids, are reported. A precatalyst approach is employed in which a matrix is prepared by incorporating M­(II) species into the pores of the sol–gel, which are later reduced to the active catalyst, M(0), during the reduction reaction by BH_4_^–^. The synthesized precatalysts are thoroughly studied using BET isotherms, X-ray diffraction, X-ray fluorescence, X-ray photoelectron spectroscopy, and scanning electron microscopy. The results point out that the relative effect of the metal-oxide environment on the adsorbed active metal species affects the process studied: for example, in the dechlorination of chloroacetic acid by Cu^0^-NPs, the order of activities is SiO_2_ > Al_2_O_3_ > TiO_2_; whereas for the analogous processes catalyzed by Ni^0^-NPs, the order of reactivities is Al_2_O_3_ > SiO_2_ > TiO_2_. For the other processes studied, other orders of activity are observed. Thus, the results indicate that the different sol–gel materials encapsulating the catalytic metals affect the reduction reactions differently.

## Graphical abstract

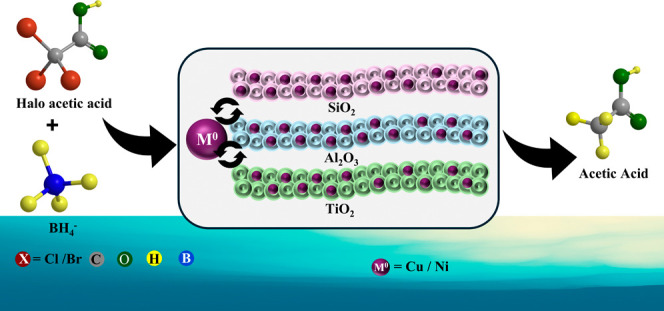

## Introduction

Today, with nanotechnology and nanoscience expanding at a rapid pace, the chemical industry and catalysis are an unbreakable duo.^1–3^ However, significant obstacles remain to be overcome, including the thermodynamic instability brought on by the high surface energy of nanoparticles and their propensity for migration and coalescence during catalysis.^4^ Currently, mesoporous inorganic oxides, metal–organic frameworks, and other architectures are being used to enhance the catalytic activity, selectivity, and recyclability of heterogeneous catalysts with encapsulated metal nanoparticles.^5–7^

In heterogeneous metal catalysts, the support’s function is typically viewed as physical, as it provides a significant surface area for forming and stabilizing nanosized metal particles. However, the idea that scattered metal particles interact with the support in a way that drastically alters the metal’s catalytic properties has long sparked interest.^8^ The supports play crucial roles, as supports often stabilize the dispersed metal particles and modify their morphology and electronic structure through strong metal–support interactions (SMSI), thereby affecting the catalyst’s performance.^9,10^ In this regard, distinct behavior was seen in terms of stability and activity in the test reaction of CO_2_ hydrogenation in a study on Co catalysts supported by Al_2_O_3_, TiO_2_, and SiO_2_. This observation was explained by the diverse chemical and electrical properties of these materials.^9^ In a similar vein, the various structural behaviors of Ni-doped on titania, alumina, and silica were also investigated for hydrogenation and steam reforming of methane.^10^ Au nanoparticles have shown higher CO oxidation catalytic activity when supported by titania, reportedly due to charge transfer at the Au/TiO_2_ interface, facilitating molecular oxygen dissociation.^11^ Similarly, Pt on TiO_2_ has shown strong metal support interactions.^8^ Most recently, the investigation into the impact of metal support interactions on the catalytic reducing properties of Au^0^ nanoparticles supported by SiO_2_ and TiO_2_ showed that the catalytic activity observed aligns with the order of TiO_2_–Au^0^-NPs > Au^0^-NPs > SiO_2_–Au^0^-NPs, suggesting that SiO_2_ donates electrons to Au^0^-NPs, while TiO_2_ withdraws them.^12^ The remarkable influence of silica support on the properties of Au^0^ and Pt^0^ nanoparticles deposited on it in catalyzing the reduction of water was studied by exploring the reaction products of the radiolytically produced (CH_3_)_2_COH^•^ radicals with the M^0^-SiO_2_ nanocomposites.^13^

These point us in the direction of possible heterogeneous catalysts, such as alumina (Al_2_O_3_), titania (TiO_2_), and silica (SiO_2_) incorporated with metal cations synthesized via the sol–gel route. The catalysts prepared are found to exhibit high surface area, purity, homogeneity, and affordability with ease of production.^14^ Sol–gel chemistry enables the entrapment of metal nanoparticles, metal complexes, enzymes, and organic species inside the matrix’s pores, while retaining their chemical activity and stability.^14^ Recent studies have shown that hybrid silica matrices prepared by the sol–gel process affect the properties of the adsorbed/entrapped species and, in some cases, even participate in chemical reactions.^15^

Although prior research has begun to explore sol–gel-based silica matrices for reductive dehalogenation,^16–18^ a systematic comparison across sol–gel-derived supports, such as titania, alumina, and silica, and their influence on the Cu and Ni nanoparticle’s behavior in the reductive dehalogenation of halo-acetic acids (HAAs), is still lacking.

In the present study, titania and alumina sol–gels were synthesized as precatalysts, an endeavor previously accomplished with silica.^16–18^ These novel precatalyst sol–gel matrices are prepared by adding transition metal salts to the precursor solution of the desired sol–gel material. These cations are retained within the forming matrix through electrostatic interactions with the deprotonated terminal hydroxyl groups, analogous to an ion-exchange mechanism. Later, these entrapped metal cations are reduced to their zerovalent state during the catalytic reduction reaction, using sodium borohydride.^16,17^ A graphical representation of this process is presented in Scheme S1. Thus, the active catalyst, M(0)@matrix, is formed, which slowly reoxidizes back to its M­(II)@matrix state after the termination of the catalytic process, thus making it reusable. This is moreover indicated by the color transitions between the characteristic hues of M­(II) and M^0^ species, during and after reactions. However, the precise characterization of these in situ formed active metal species, presumably M^0^ nanoparticles, remains challenging due to their confinement within the sol–gel pore structure and their instability due to their reoxidation to the M­(II) state. The formed M^0^ species are similar but not identical to presynthesized and encapsulated metal nanoparticles as reported earlier.^16^ This can be explained by differences in size and/or morphology between the presynthesized nanoparticles and those formed in situ. These precatalyst versions of Cu and Ni encapsulated inside silica, alumina, and titania are hereafter designated as 1% Cu­(II)@SiO_2_, 1% Cu­(II)@Al_2_O_3_, 1% Cu­(II)@TiO_2_, 1% Ni­(II)@SiO_2_, 1% Ni­(II)@Al_2_O_3_, and 1% Ni­(II)@TiO_2_, respectively.

The second most common disinfection byproducts (DBPs) produced in treated water are HAAs, which are cytotoxic and genotoxic.^19^ Water remediation and removal of toxic DBPs are a dominant field of interest for scientists these days. DBPs can be reduced through advanced oxidation processes, membrane filtration, and optimized conventional pretreatments that lower natural organic matter before disinfection. Additional strategies include adjusting chlorine dosing, using alternative disinfectants, post-treatment methods (adsorption and biofiltration), and applying AI-driven process optimization to enhance safety and sustainability.^20^

Here, the catalytic efficiency in dehalogenating the toxic DBPs tribromoacetic acid (TBAA), monobromoacetic acid (MBAA), trichloroacetic acid (TCAA), and monochloroacetic acid (MCAA) was tested. Our previous work demonstrated the impact of metal nanoparticle’s nature on the dehalogenation of HAAs.^17^ In this study, the focus is on another intriguing aspect: the effect of different metal-oxide-based sol–gel matrices on the catalytic activity of the entrapped/adsorbed metal species in their pores.

## Experiments and Methods

### Materials

Titanium­(IV)-isopropoxide (TTIP) (≥97%), aluminum-isopropoxide (AIP) (≥98%), CuCl_2_·2H_2_O (99%), tribromoacetic acid (TBAA) (97%), dibromoacetic acid (DBAA) (96%), monobromoacetic acid (MBAA) (98+%), tetraethyl-orthosilicate (TEOS) (≥99%), methyl-trimethoxy-silane (MTMOS) (≥99.0%), and APS (3-aminopropyl-triethoxysilane) were purchased from Sigma-Aldrich (St. Louis). NiCl_2_·6H_2_O (>98%), trichloroacetic acid (TCAA) (99%), and monochloroacetic acid (MCAA) (99%) were purchased from Alfa Aesar (Ward Hill, MA). Acetic acid (AA) (glacial, 99.7%), fumaric acid (FA) (99%), and NaBH_4_ powder (99%) were purchased from Thermo Scientific (USA). Hydrochloric acid (HCl, 37%), HPLC grade 85% H_3_PO_4_, maleic acid (MA), and LC–MS grade ethanol were purchased from Merk (Germany). 30% NH_3_ solution was obtained from Carlo Erba reagents (France) and nitric acid (HNO_3_) (70%) from Daejung Chemicals and Metals Co., Ltd. (Korea). Acetonitrile (LC–MS grade) was from Bio Lab Ltd. (Israel). All chemicals were of A.R. grade and were used as received. All aqueous solutions were prepared from deionized water purified by a Millipore Milli-Q setup with a final resistivity of >10 MΩ/cm.

### Instrumentation and Measurements

The Brunauer–Emmett–Teller (BET) isotherms and analysis helped to confirm the mesoporous nature of the catalyst matrices. BET measurements for the determination of specific surface area, pore volume, and pore size distribution were conducted utilizing a Micromeritics Tristar II Plus analyzer using N_2_ adsorption at 77 K. Before analysis, the samples underwent a 24 h degassing at 110 °C under vacuum. The surface area was derived from the linear segment of the BET plot, while the pore size distribution was assessed employing the Barrett–Joyner–Halenda (BJH) model and the Halsey equation at *p*/*p*_0_ ∼ 0.98.

SEM images, along with elemental composition assessment by energy-dispersive X-ray spectroscopy (EDS), were taken to understand the surface morphology of the catalysts synthesized. The analysis was performed on an Ultra-High Resolution MAIA 3 FE-SEM (Tescan) with Microanalysis EDX Aztec (Oxford) with X-MAX^N^ active area 80 mm^2^ and resolution 127 eV.

Phase identification and a deeper understanding of the sample purity were achieved through powder X-ray diffraction analysis with an X-ray generator at 40 kV using the ICDD PDF2 2019 database. Using the Rigaku SmartLab II (v4.5.270.0) SE diffractometer, the reference intensity ratio (RIR) method and figure of merit (FOM) calculation were used for data analysis. Raman spectroscopy measurements were performed by using a Witec360 Raman microscope with a laser wavelength of 532 nm.

X-ray photoelectron spectroscopy (XPS) characterization was performed using an “ESCALAB Xi+” by Thermo Fisher Scientific. The ambient pressure in the chamber is <1 × 10^–10^ mbar. At room temperature, photoelectron emission was achieved by using an Al Kα radiation source (1486.68 eV). The spectra were collected at 90° from the X-ray source. A low-energy electron flood gun was used to minimize the charge at the surface. The powders were also pressed into an indium foil to further minimize the charging and their movement. Measurements were taken with a spot diameter of 650 μm, where no indium was observed in the survey spectrum. Avantage software version 6.6.0. by Thermo Scientific was used for the XPS spectra processing. A high-resolution spectrum was measured for each relevant element, along with a survey spectrum.

X-ray fluorescence wavelength dispersive (WDXRF) Spectrometer Axios (1 kW) with SuperQ version 5 software, PANalytical B.V., Almelo, The Netherlands, was used for XRF measurements. The special software Omnian, based on the fundamental parameter method, was used for quantitative analysis. The samples were analyzed as loose powders. Inductively coupled plasma-optical emission spectroscopy analysis was conducted using a Thermo Scientific iCAP PRO IQ instrument.

Dehalogenation of the HAAs was monitored by using an HPLC system (Dionex Ultimate 3000) with a UV/visible detector (λ = 210 nm) along with an Agilent HPLC column (Eclipse XDB-C18, 3 μm) with dimensions of 4.6 × 150 mm. The eluent was H_2_O: acetonitrile; 98:2 with 0.2% *ortho*-phosphoric acid (H_3_PO_4_), maintaining pH between 2 and 3, a flow rate of 1 mL/min, and 25 °C column oven temperature.

### Syntheses

The synthetic procedures adapted for preparing the different catalysts are described herein (Scheme 1). The metal content was kept constant as a 1 mol percentage of the total metal-alkoxide (TiO_2_/Al_2_O_3_/SiO_2_) content. The heat treatment conditions were optimized to minimize metal leaching from the metal-oxide matrix.

**Scheme 1:**
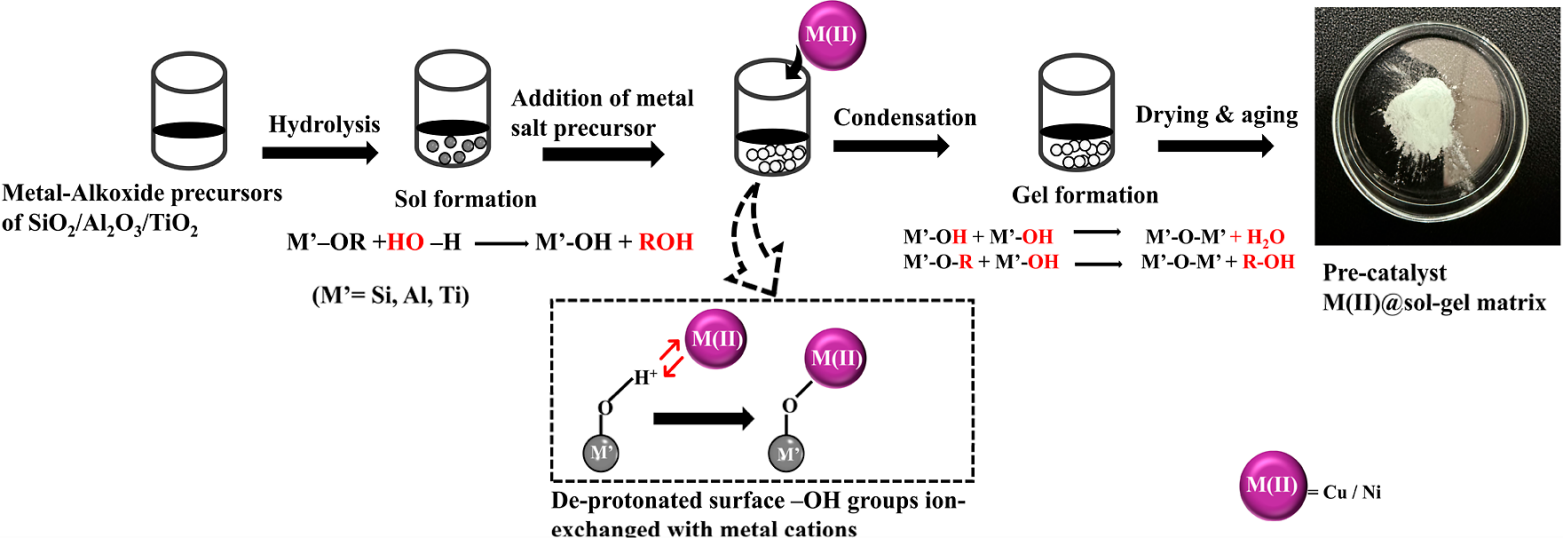
Schematic Representation of the Pre-Catalyst Synthesis via the Sol–Gel Method^a^

a Photograph shown is that of Cu­(II)@Al_2_O_3_.

#### 1% M­(II)@TiO_2_

The precatalyst preparation was inspired by the works of Gonçalves et al.^10^ Initially, at room temperature, the mixture of ethanol and TTIP (titanium­(IV)-isopropoxide) was thoroughly homogenized, followed by the addition of a required metal salt precursor (CuCl_2_·2H_2_O/NiCl_2_·6H_2_O). After it was completely dissolved, acid hydrolysis was initiated using a 1.67 M HNO_3_ solution, and instantaneous gel formation occurred. The final molar ratio of *n*_water_/*n*_alkoxide_/*n*_acid_ was 2.4:1:0.08. After aging and drying for 7 days, the catalyst was heated at 200 °C for 24 h. Then the powdered catalyst was washed with water and dried again at 40 °C before the catalytic tests.

#### 1% M­(II)@Al_2_O_3_

The alumina matrices were prepared following a procedure reported in the literature.^21^ Briefly, 8.16 g of aluminum-isopropoxide (AIP) was hydrolyzed using 48 mL of water maintained at 90 °C. To this, CuCl_2_·2H_2_O/NiCl_2_·6H_2_O was added, constituting 1 mol percentage of the alumina. To the homogeneous solution, 4 mL of 0.89 M HNO_3_ solution was added and stirred for 48 h at 90 °C. The sol was condensed by evaporation, and the resulting gel was calcined at 600 °C for 5 h. Later, the powdered gel was washed and dried before use in catalytic experiments.

#### 1% M­(II)@SiO_2_

The silica-based catalysts were prepared following the conventional pathway from our lab as reported earlier.^17^ We employed a two-step acid–base sol–gel synthesis method using tetraethyl-orthosilicate (TEOS) and methyl-trimethoxy-silane (MTMOS) as the alkoxide precursors in a molar ratio of 70:30. After the thorough mixing of the precursors, a 0.26 M HCl solution was added for acid hydrolysis, followed by the addition of the required metal salt and 1.5 mL of a 2% NH_3_ solution that catalyzes the gelation process. The obtained gel is kept for aging and drying for nearly 14 days at room temperature, and then powdered and washed with water before use. Blank matrices were prepared following the same procedure without the addition of the metal salts.

### Catalytic Tests

For the catalytic tests, in a 50 mL centrifuge tube, the required substrate solutions (0.05 M) were mixed with a suspension of the catalyst in deionized water (the exact amount of catalyst used is provided specifically for each reaction in the caption of the results). The reactions were conducted at room temperature, with an initial pH range between 1 and 2. After the mixture was stirred for a short duration, the reducing agent (NaBH_4_ powder) was gradually added, leading to a change in pH to a range of 8–9. The ratio between [substrate]:[NaBH_4_] varies depending upon the number and strength of C–X bonds (exact concentration and amount are provided in the captions to the result’s figures). The resulting suspension was stirred for a total of 60 min in the case of TBAA and MBAA reduction, and 5 h for TCAA and MCAA reduction. The reaction time is also varied due to the stronger C–Cl bond strength compared to the C–Br bond.^22^ Subsequently, the catalyst was separated by centrifugation, and the filtrate was subjected to HPLC analysis. After each cycle, the catalyst is washed using milli-Q water followed by centrifugation at 1000 rpm for 15 min. Each experiment was repeated a minimum of three times, and the reusability of the catalyst matrices was confirmed for up to three cycles. The error limit of the presented analytical results is ±5%.

## Results and Discussion

### Precatalyst Characterization

Figure S1 shows the SEM images of the precatalysts studied. The surface morphology identifies the presence of elements in the metal-oxide matrix and shows hardly any presence of the encapsulated metal species. This proves that the active metal cations are well encapsulated inside the sol–gel structures and are not scattered on their surfaces. The same results were obtained when examined after a cycle of reaction, thus pointing toward the stability of the surface morphology of the precatalysts/catalysts.

Table 1 presents the surface characteristics derived from the slopes and intercepts of the linearized nitrogen adsorption isotherms and pore sizes derived from the BJH pore-distribution plots, providing key textural parameters of the investigated precatalyst matrices. The BET surface area analysis revealed that alumina sol–gel has the lowest surface area, which increases after adding the respective metal salt. This lowered surface area and larger pore size than titania-based sol–gels can be explained by the higher calcination temperature used for its synthesis, as there is a chance for decreasing surface area and increasing pore sizes with increasing temperature.^23^ The increase in the pore volume of the catalysts with the incorporation of metal cations can be due to the disruption of the regular packing of mesoporous sol–gel structures by metal cations with different charges and ionic radii. The change in pH and gelation kinetics due to metal addition affects the hydrolysis and condensation stages, thus disrupting the sol–gel skeleton, leading to larger pore sizes.^24^

**Table 1:** BET Analysis Results

pre-catalyst	surface area (m^2^/g)	average pore radius (Å)	pore volume (cm^3^/g)
Al_2_O_3_	181	26.2	0.29
1% Cu(II)@Al_2_O_3_	215	28.4	0.38
1% Ni(II)@Al_2_O_3_	213	26.9	0.36
TiO_2_	269	16.6	0.25
1% Cu(II)@TiO_2_	434	13.9	0.32
1% Ni(II)@TiO_2_	470	12.5	0.28
SiO_2_ (ORMOSIL)^16^	640	11.4	0.37
1% Cu(II)@SiO_2_ ^17^	518	10.4	0.27
1% Ni(II)@SiO_2_ ^18^	602	18.0	0.34

Figures S2 and S3 show the BET isotherms obtained for the blank matrices versus those of the metal-encapsulated matrices and the corresponding BJH pore-distribution curves. As per the IUPAC classification, except for the M­(II)@TiO_2_, these correspond to the type IV isotherms with an H_2_-hysteresis loop, which are characteristic of mesoporous adsorbent materials.^25,26^ However, an H_4_ slit-like hysteresis-type loop was exhibited by metal-encapsulated titania matrices, similar to previously reported cases.^27^

Figures 1–4 show the X-ray diffraction peaks obtained for the precatalysts employed, in comparison to the XRD pattern obtained for the blank alumina and titania. Alumina-based precatalysts exhibited the γ-Al_2_O_3_ phases at angles 37.62°, 45.8°, 60.7°, 66.74°, and 84.7° corresponding to the (3 1 1), (4 0 0), (5 1 1), (4 4 0), and (4 4 4) planes, respectively (card number: 01-079-1558). One can see that these peaks consistently appear in all matrices containing alumina. Due to the very small percentage (1 mol %) of the metal cations inside the sol–gel matrices, it is hardly detectable in the XRD pattern, but a closer examination of the peaks shows the following data: the phases of CuCl_2_·2H_2_O can be seen for 1% Cu­(II)@Al_2_O_3_ as (1 0 1), (2 0 0), (0 1 2), (0 0 4), and (3 1 3) planes (card number: 01-077-9005). In the case of Ni@Al_2_O_3_, the syn-phases of bischofite (NiCl_2_·6H_2_O) corresponding to (1 0 2), (0 2 0), (2 1 1), (2 2 4), (2 3 5), and (0 4 3) were seen (card number: 01-072-2027) (Figure 1). (Note that the diffraction patterns of Cu and Ni are so close that they cannot be easily differentiated.)

**Figure 1:**
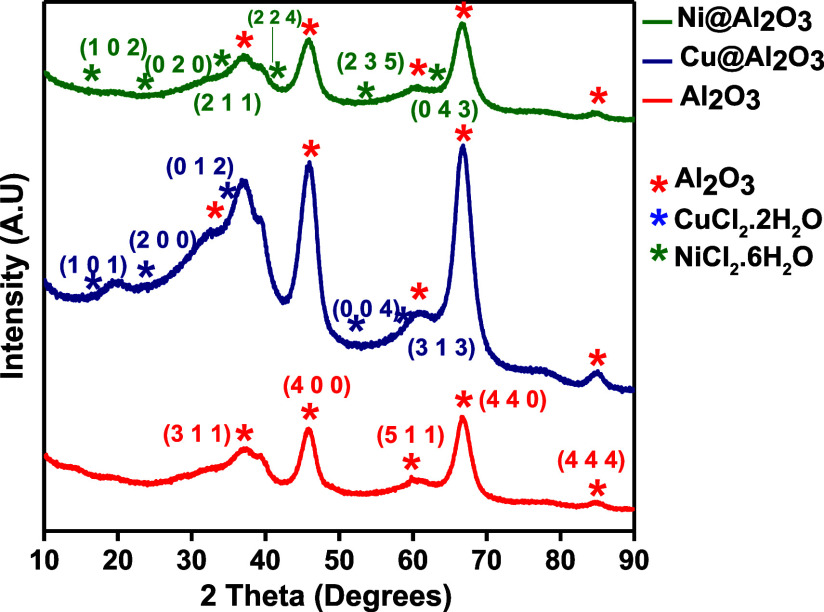
XRD spectra of alumina (Al_2_O_3_), 1% Cu­(II)@Al_2_O_3_, and 1% Ni­(II)@Al_2_O_3_ (before reduction reaction).

XRD analysis of these catalysts was also conducted after a cycle of reaction (Figure 2). It was seen that the Cu@Al_2_O_3_ catalyst retained almost all the phase characteristics in addition to some new peaks corresponding to the phases of syn Al­(OH)_3_ (card number: 01-077-9948). This can be attributed to the formation of a hydrated layer of hydroxide (Al­(OH)_3_) at the surface of γ-Al_2_O_3_ after it is suspended in aqueous solutions at pH > 4, possibly by the γ-Al_2_O_3_ surface hydration through hydrolysis of surface Al–O bonds.^28^ In the case of Ni@Al_2_O_3_, after a reaction cycle, significant phase changes were observed due to the involvement of NaBH_4_ and predominant peaks of syn borax (Na_2_H_20_B_4_O_17_) (card number: 01-074-0339) and syn tincalconite (Na_2_[B_4_O_5_(OH)_4_]·3H_2_O) (card number: 00-007-0277).

**Figure 2:**
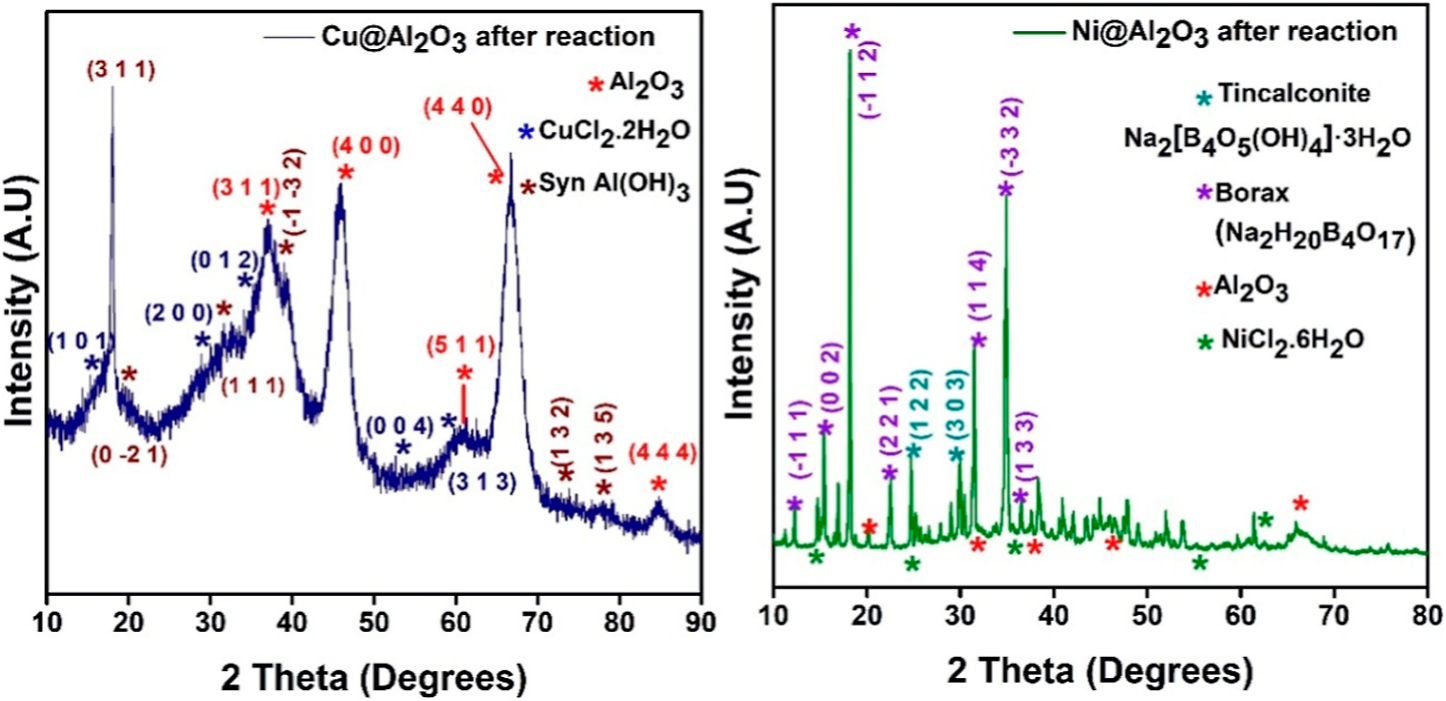
XRD spectra of 1% Cu­(II)@Al_2_O_3_ and 1% Ni­(II)@Al_2_O_3_ (after reduction reaction).

Titania sol–gels exhibited both anatase and rutile syn phases, as was expected.^10^ Approximately 99.5% consists of the rutile phase, the most stable phase of TiO_2_, while the remaining 0.5% is the anatase phase. The anatase phases of TiO_2_ were observed at 2θ degrees of 25°, 38.6°, 62.2°, 69.3°, 70.2°, and 82.8° corresponding to the (1 0 1), (1 1 2), (2 0 4), (1 1 6), (2 2 0), and (2 2 4) planes, respectively (card number: 01-075-2553). While the rutile phases were observed at angles 27.4°, 36.1°, 41.24°, 44.06°, 54.33°, 56.64°, 64.07°, 69.01°, 72.43°, 84.27°, and 87.5° corresponding to the (1 1 0), (1 0 1), (1 1 1), (2 1 0), (2 1 1), (2 2 0), (3 1 0), (3 0 1), (3 1 1), (4 0 0), and (4 1 0) planes (card number: 00-021-1276), respectively. These phases are also retained in the Ni­(II) and Cu­(II) encapsulated titania sol–gels, except that the anatase phase was absent in the case of Cu­(II)@TiO_2_. For Ni­(II)@TiO_2_, nickel-titanate (Ni­(TiO_3_)) phase, a coordination complex between Ni­(II) and Ti­(IV),^29^ was observed at 24.12°, 33.1°, 35.81°, 41.25°, 43.57°, 49.45°, 53.98°, 62.31°, and 69.52° corresponding to the (0 1 2), (1 0 4), (1 1 0), (1 1 −3), (2 0 2), (0 2 4), (1 1 −6), (1 2 −4), and (2 0 8) planes, respectively (card number: 01-077-0152). In the case of Cu­(II)@TiO_2_, a syn form of CuO called tenorite were observed at 2θ angles 32.5°, 35.5°, 38.7°, 48.7°, 61.5°, 68.1°, 72.4°, and 80.2° corresponding to the (1 1 0), (1 1 −1), (1 1 1), (2 0 −2), (1 1 −3), (2 2 0), (3 1 1), and (2 0 −4), respectively (card number: 00-045-0937) (Figure 3).

**Figure 3:**
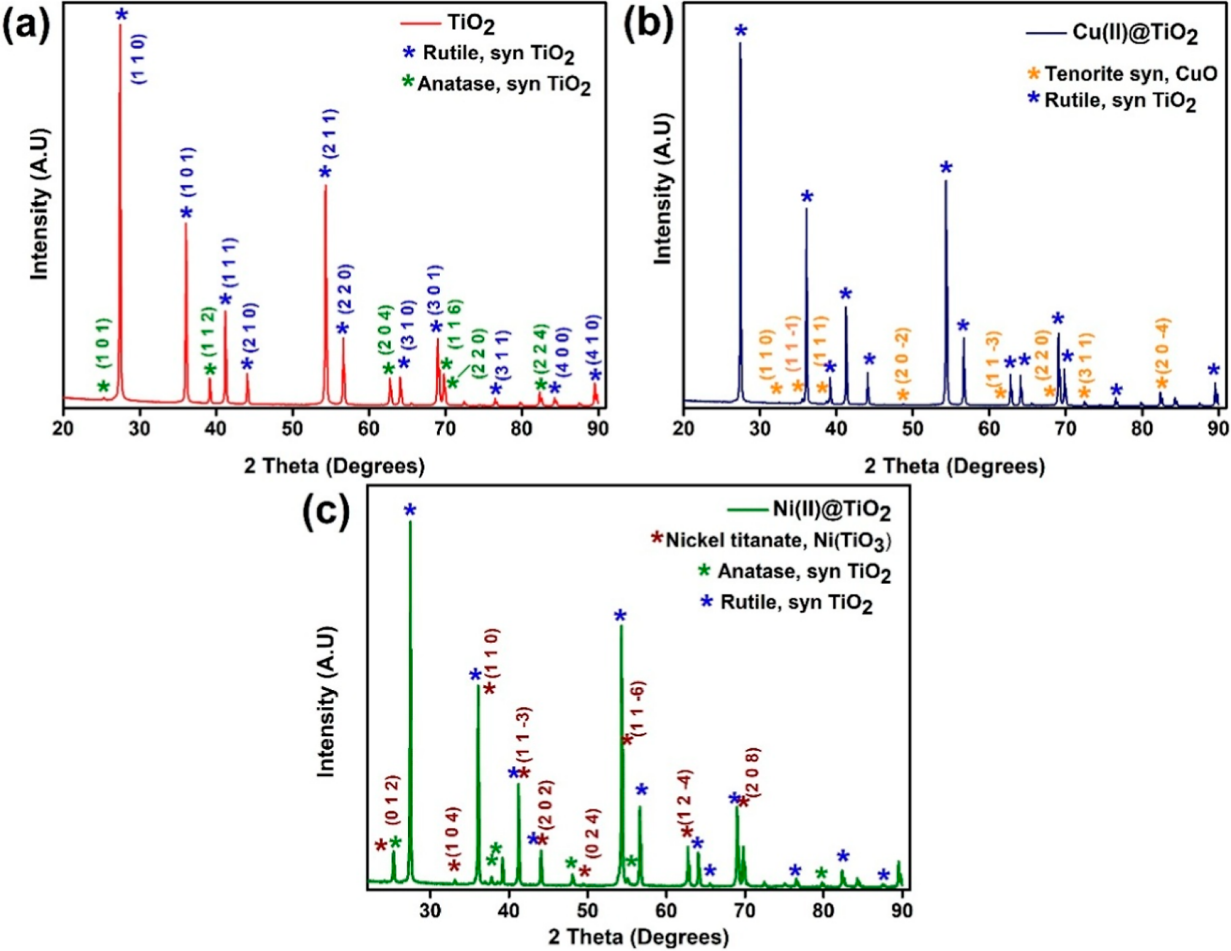
XRD spectra of (a) TiO_2_, (b) 1% Cu­(II)@TiO_2_, and (c) 1% Ni­(II)@TiO_2_ (before reduction reaction).

Examining the XRD peaks after a reaction cycle reveals that both the anatase and rutile phases of TiO_2_ that existed before the reaction more or less disappeared and possibly transformed to the brookite form. However, the phases of nickel titanate and CuO are retained. Furthermore, the peaks are broader and noisier, possibly due to the NaBH_4_ reduction, which can lead TiO_2_ to undergoing crystalline lattice deformation, resulting in oxygen vacancies, and amorphous TiO_2_ can be formed^30^ (Figure 4). These reasons can also account for the complexity in the phase changes. Again, the weak peak intensities of the metal phases are possibly due to their very low percentage (1%) of encapsulation inside the matrices, which is below the detection limit of XRD.

**Figure 4:**
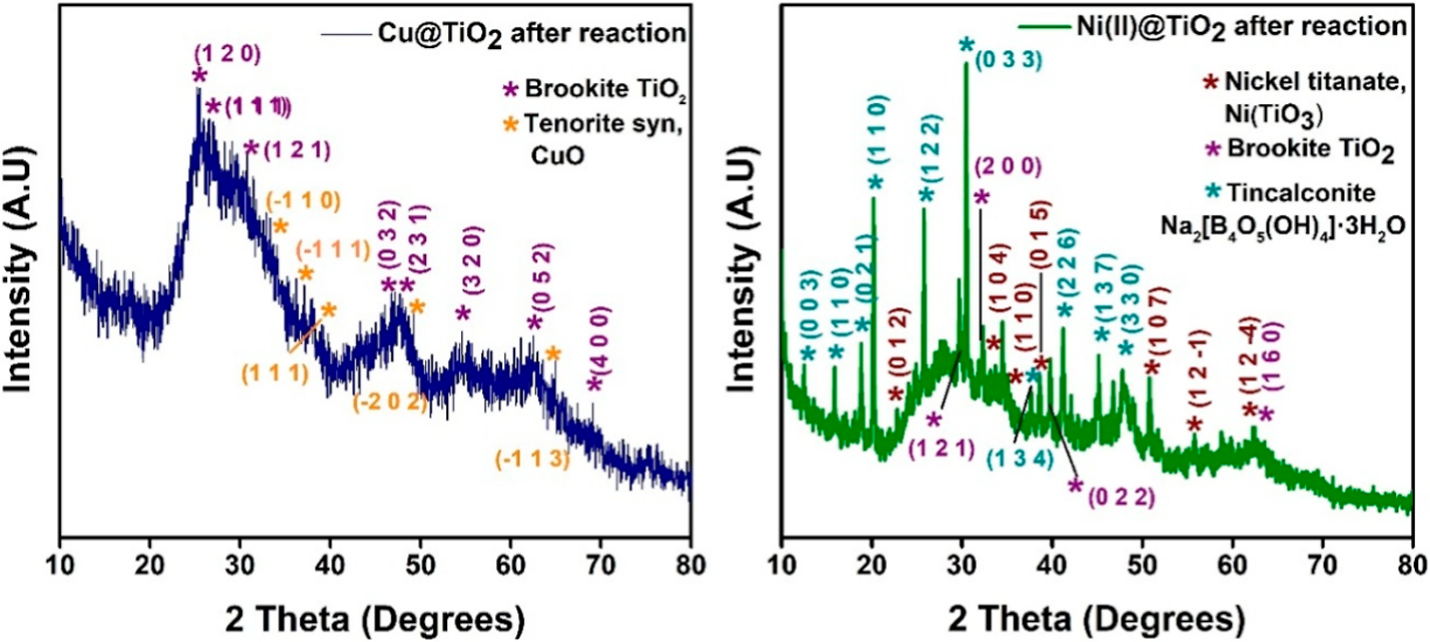
XRD spectra of 1% Cu­(II)@TiO_2_ and 1% Ni­(II)@TiO_2_ (after reduction reaction).

To evaluate the possible phase transitions in titania during catalysis, Raman analyses were performed for the postreaction catalyst and compared with the spectra recorded before catalysis. Though XRD provides long-range crystallographic fingerprints, Raman spectroscopy offers superior sensitivity toward structural distortions, making it effective for distinguishing among TiO_2_ polymorphs. As shown in Figure S4a,b, the Raman spectra of the blank TiO_2_ matrix and Cu­(II)@TiO_2_ exhibit four characteristic Raman active modes of the rutile TiO_2_ phase. The prominent vibrational modes corresponding to the B_1g_, E_g_, and A_1g_ symmetries appearing at 142, 442, and 610 cm^–1^, along with the multiple phonon-scattering process at ∼232 cm^–1^, confirm the rutile phase.^31^ Notably, the Raman spectrum of Cu­(II)@TiO_2_ after one reaction cycle (Figure S4c) indicates that the rutile phase undergoes a structural transformation during the reaction. To identify the individual vibrational modes distinctly, the Raman spectrum was deconvoluted using Lorentzian curve fitting and is presented in the inset of Figure S4c. The deconvoluted spectrum displays six phonon modes at approximately 153, 210, 250, 413, and a split band near 637 cm^–1^ corresponding to the characteristic A_1g_ and B_1g_ vibrational modes of the brookite phase.^32,33^ This distinct spectral change suggests that the catalytic process triggers a rutile-to-brookite phase transition in the Cu­(II)@TiO_2_ system, in agreement with the XRD results. However, one cannot neglect the fact that trace amounts of the rutile phase may still be present that remain undetectable by Raman/XRD.

Likewise, the fresh Ni­(II)@TiO_2_ catalyst predominantly exhibited the anatase phase, characterized by its typical vibrational modes E_g_, B_1g_, A_1g_, and E_g_, located at 143, 393, 512, and 637 cm^–1^, respectively.^31,34^ After one reaction cycle, the used catalyst loses this phase completely or partially and may be transformed into the brookite phase, displaying the same characteristic bands previously observed for the used Cu­(II)@TiO_2_ sample (Figure S5). This result further indicates the possibility of a structural transition as suggested by the XRD analysis.

The elements found on the surfaces of the catalysts and their oxidation states were analyzed by using XPS. It is important to note that the photoelectron spectroscopy results reveal the composition of the surface and the near-surface region.^35^ The typical full-scan spectra disclose the presence of Al, Ti, Cu, Ni, O, and C in the precatalysts (Figure S6).

From the XPS spectra of fresh Cu precatalysts, the binding energy for copper (Cu 2p_3/2_) is observed at around 933.8 eV in the case of Cu­(II)@Al_2_O_3_ and at 932.9 and 942.37 (Cu^2+^ in CuO) for Cu­(II)@TiO_2_, both characteristic of Cu^2+^ peaks closely aligning with earlier reported studies^36,37^ (Figure 5). The positive binding energy shift in the former suggests a stronger charge transfer between Cu and Al_2_O_3_ than between Cu and TiO_2_. For Cu^2+^ (d^9^), satellite structures/shakeup bands are present, as seen in the spectra between 933.5 and 942.6 eV.^38,39^ These satellite peaks exclusively identify Cu^2+^, as charge transfer cannot occur in Cu^+^ compounds or Cu^0^ species due to their filled 3d shells.^40^ For the used Cu­(II)@TiO_2_ catalysts, a peak at 932.4 and the disappearance of satellite peaks indicate the presence of Cu^+^ in Cu_2_O. These shifts suggest a possible reduction of Cu^2+^ to Cu^+^ or Cu^0^ (the difference between Cu^0^ and Cu^+^ can be 0.1 eV^41^). This reduction is further evidenced by notable color changes (blue to dark brown), which reverse over time. The presence of mixed oxidation states of Cu, although the source of Cu was the Cu­(II) salt, has been reported.^39,42^ The shift of binding energy from 933.6 eV suggests the substitutional incorporation of Cu ions in the lattice of TiO_2_/Al_2_O_3_.^43^

**Figure 5:**
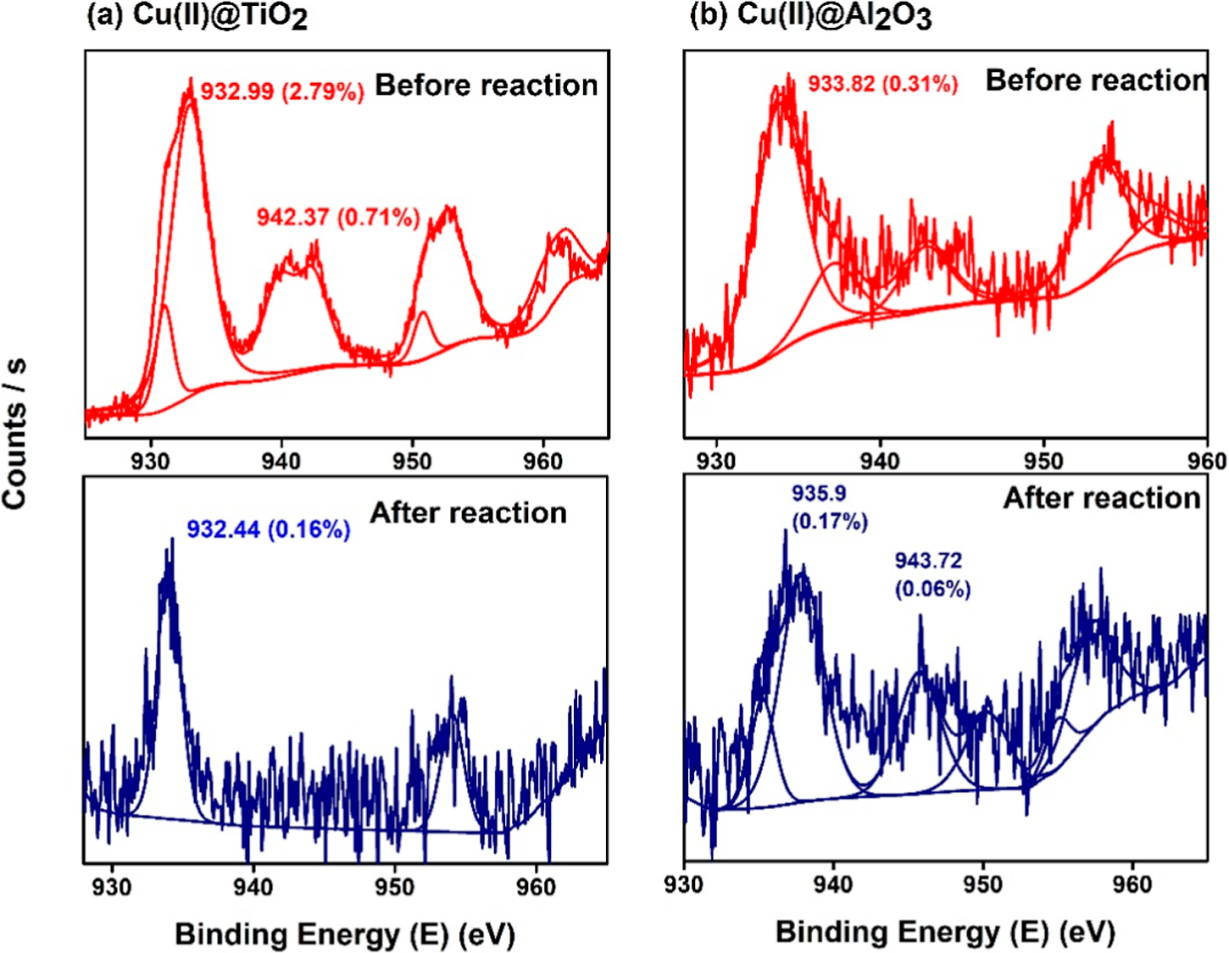
XPS spectra of Cu 2p_3_ from (a) Cu­(II)@TiO_2_ and (b) Cu­(II)@Al_2_O_3_ analyzed before and after a cycle of reaction.

In the case of Ni­(II)@Al_2_O_3_, the binding energy of Ni 2p_3_ is at 857.28 eV and Ni­(II)@TiO_2_ at 856.53 eV (Figure 6). These correspond to the characteristic binding energies of Ni^2+^ appearing between 855.2 and 857 eV with the characteristic satellite/shakeup bands around 860.7 eV.^44^ Like in Cu^2+^ compounds, shakeup bands where a valence electron from the ligand is excited to an unoccupied metal orbital (from np_L_ to 3d_M+_), alongside the main core photoelectron process, are seen here as well. Consequently, information is gathered not only from binding energy values but also from the positions of the satellite peaks.^45^ The peaks around 855.1 may indicate the bonding of Ni^II^–O and 856.7 to Ni^II^–Cl.^46^ But the absence of Cl peaks rules out the chances of the latter. In addition, spectra of Ni­(II)@TiO_2_ after the reaction show no satellite peaks signaling the Ni^2+^ state and revealing that Ni^2+^ is very probably doped into the TiO_2_ lattice by replacing Ti^4+^.^47^ This idea is strengthened with the O 1s spectra shifting to the 532.02 eV region, indicating the oxygen vacancies formed after the reduction of Ti^4+^ by NaBH_4_ and thus the substitution by Ni^30^ (Figure 7). This is in agreement with the XRD results reported above. A similar observation is seen with the Ni­(II)@Al_2_O_3_ used catalyst. The faint nature of the metallic peaks suggests that most metallic species are situated within the inner pores of the matrices rather than on the catalyst’s surface.

**Figure 6:**
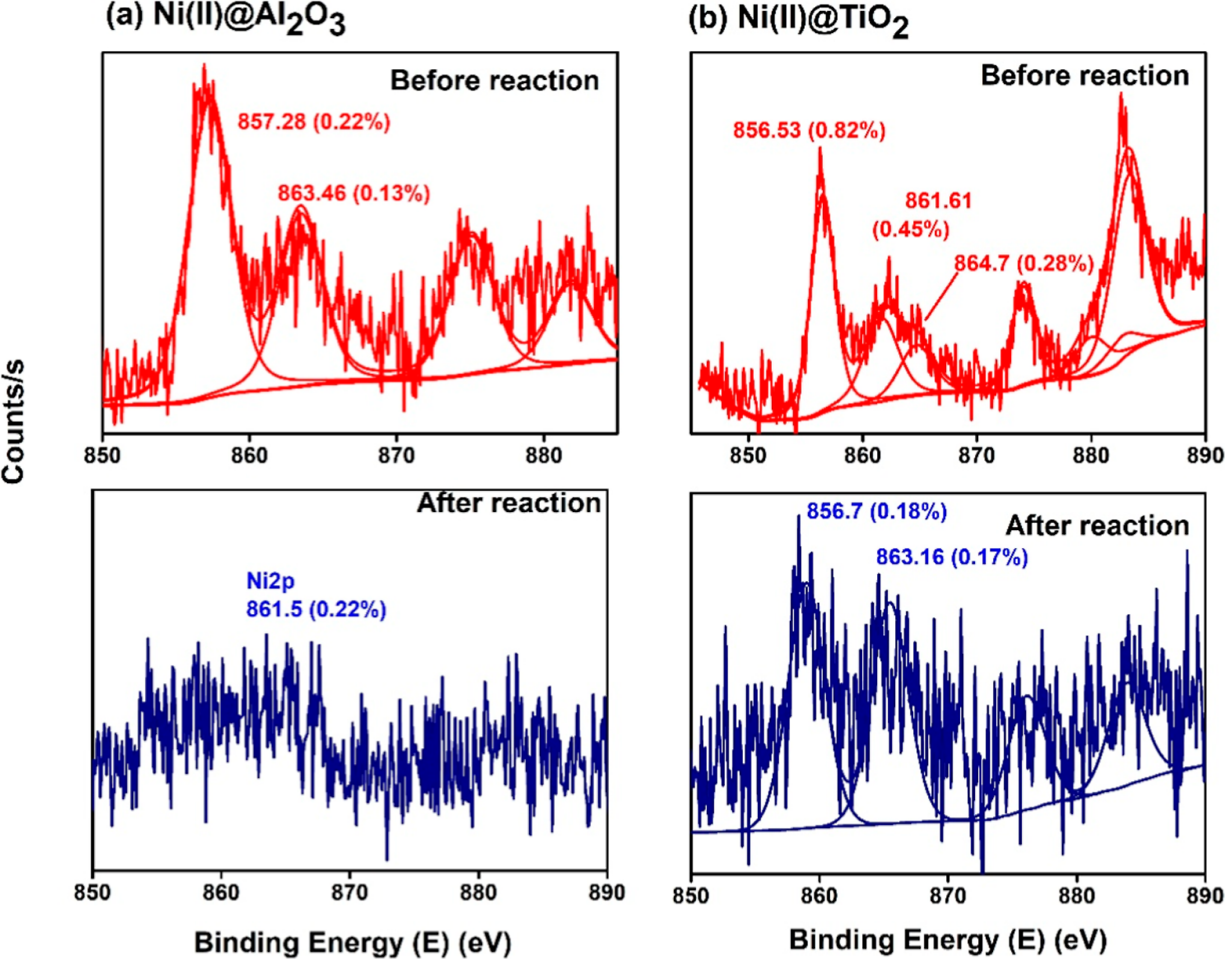
XPS spectra of Ni 2p_3_ from (a) Ni­(II)@Al_2_O_3_ and (b) Ni­(II)@TiO_2_ analyzed before and after a cycle of reaction.

**Figure 7:**
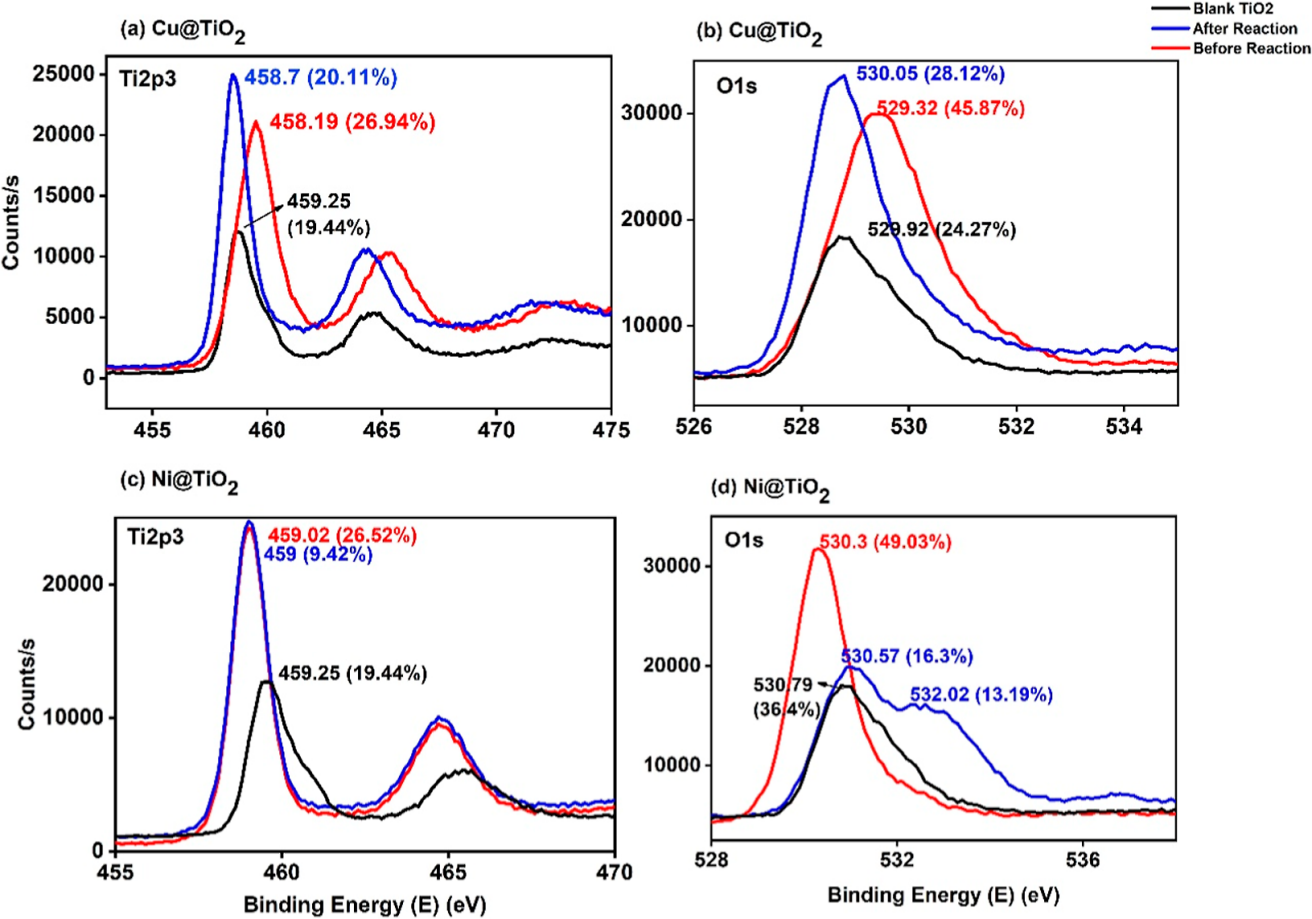
Ti 2p and O 1s XPS spectra of (a) and (b) Cu­(II)@TiO_2_ and (c) and (d) Ni­(II)@TiO_2_, respectively.

The γ-Al_2_O_3_ shows an Al 2p oxide peak at 73.23 eV, which is around the reported data,^48^ and one can see shifts in this binding energy to 74.43 eV (Cu­(II)@Al_2_O_3_) and 74.48 eV (Ni­(II)@Al_2_O_3_) attributed to a certain degree of interaction with the adsorbed cations leading to charge transfer between the matrix and metal.^49–51^ The shifting of binding energy after a cycle of reaction can also be explained due to the formation of Al­(OH)_3_, which can also be seen in the XRD data.^29^ The deconvoluted O 1s spectrum shows three characteristic peaks in the range of 530.1 to 532.4 eV, representing the three forms of oxygen possible: lattice oxygen present in TiO_2_ and Al_2_O_3_; due to OH groups; peaks from both organic carbon contaminants and H_2_O and low coordinated oxygen species due to doping.^48,52^ The O 1s peaks around 532 eV represent the O–H bonding and/or oxygen vacancies,^30^ and the lower binding energy indicates Cu–O or Ni–O bondings.^53^ The clear presence of Ti^4+^ species from TiO_2_ can be observed in all the precatalysts based on titania with binding energies around 459–458 eV as reported in the literature.^43,48^ After encapsulating with the cations, the shift of Ti^4+^ peaks to lower binding energies can be explained by the formation of Ti^3+^ or the substitution of Ti^4+^ by Cu^2+^/Ni^2+^ ions^43^ (Figures 7 and 8).

**Figure 8:**
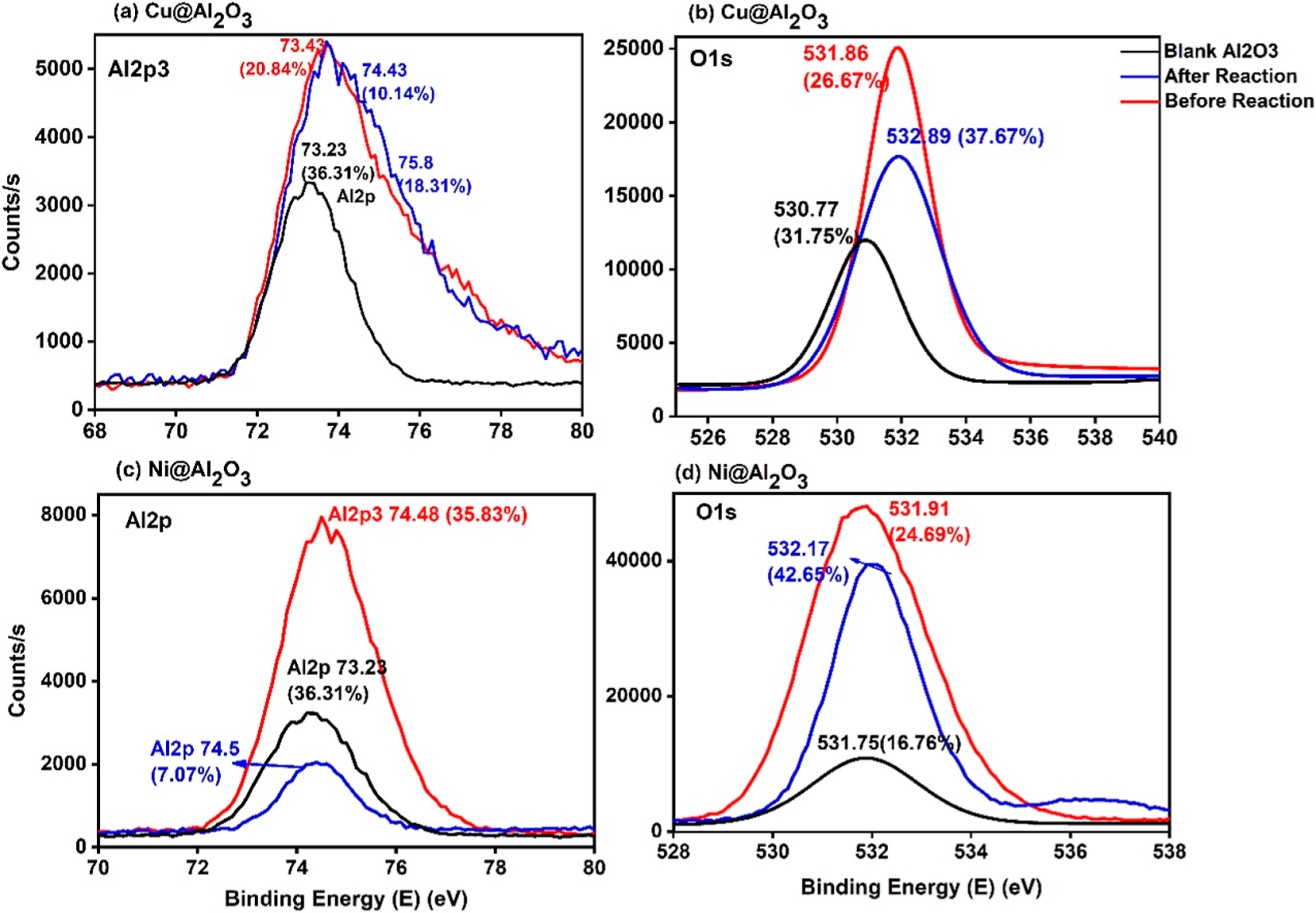
Al 2p and O 1s XPS spectra of (a) and (b) Cu­(II)@Al_2_O_3_ and (c) and (d) Ni­(II)@Al_2_O_3_, respectively.

To further elucidate, XPS data on the atomic percentages of each sample are helpful (Table S1). The comparatively lower presence of metal species in alumina than in titania suggests that alumina encapsulates more metal species. At the same time, some aggregation occurs on the surface of titania. This is supported by XRF results showing a higher metal percentage in the bulk of alumina than in titania. XRF analysis confirmed the presence of the target metal species within the catalyst bulk; however, the precision of the exact mol % determined by XRF is subject to debate due to the relatively low metal content. The catalysts with their corresponding metal loadings from the XRF analysis are listed in Table S2. XPS analysis on silica surfaces showed hardly any presence of Cu and Ni, indicating stronger encapsulation by silica. Therefore, ICP analysis of silica-encapsulated catalysts by forced leaching using HNO_3_ yielded results indicating the metal encapsulation (Table S3).

The binding energy results indicate a larger electron transfer from the titania to the entrapped M­(II) cations than that from the silica. This is opposed to the trend reported for the electron transfer from the analogous matrices to Au^0^-NPs.^12^ This might be due to the different charges of the M^0^ and the M­(II).^54^

The detailed characterization and catalytic test results of silica-based catalysts were already reported.^17^

### Catalytic Activity and Mechanisms

Catalytic efficiency experiments in reducing MCAA, TCAA, TBAA, and MBAA were conducted.

#### MCAA Reduction Reaction

Among the HAAs studied herein, MCAA is the most difficult to dehalogenate, making it the most suitable reference for studying the varying efficiencies between different catalyst systems. The order of MCAA reduction, Figure 9, follows the order: Ni­(II)@Al_2_O_3_ (65%) > Cu­(II)@SiO_2_ (55%) > Cu­(II)@Al_2_O_3_ (41%) > Ni­(II)@SiO_2_ (28%) > Ni­(II)@TiO_2_ (21%) > Cu­(II)@TiO_2_ (12%). So, for Cu, the order of activity of the metal-oxide systems is SiO_2_ > Al_2_O_3_ > TiO_2_. While for Ni, it is Al_2_O_3_ > SiO_2_ > TiO_2_. In both cases, the catalysts incorporated in titania matrices show the lowest reduction efficiency. The reusability of the catalysts was tested for three consecutive cycles (Figure 10), showing that there is a ∼10% decrease in activity for the Ni@Al_2_O_3_ and ∼20% drop in the activity for Ni@TiO_2_ catalysts, possibly explained by the XPS results showing the depletion of Ni^2+^ from the surface after the first cycle of use.

**Figure 9:**
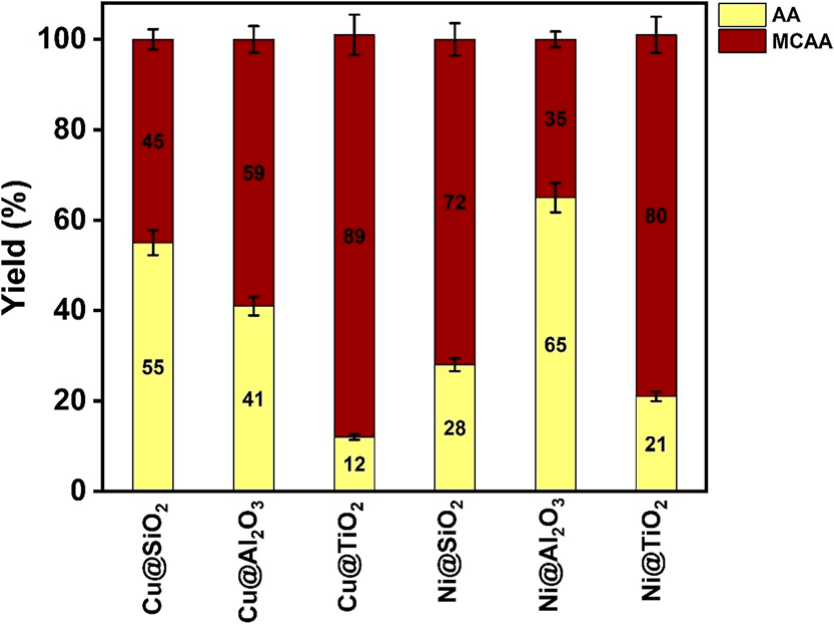
Product distribution in % of MCAA reduction. (Catalyst amount 0.5 g, [MCAA] = 0.05 M, [NaBH_4_] = 0.75 M, 0.43 g.)

**Figure 10:**
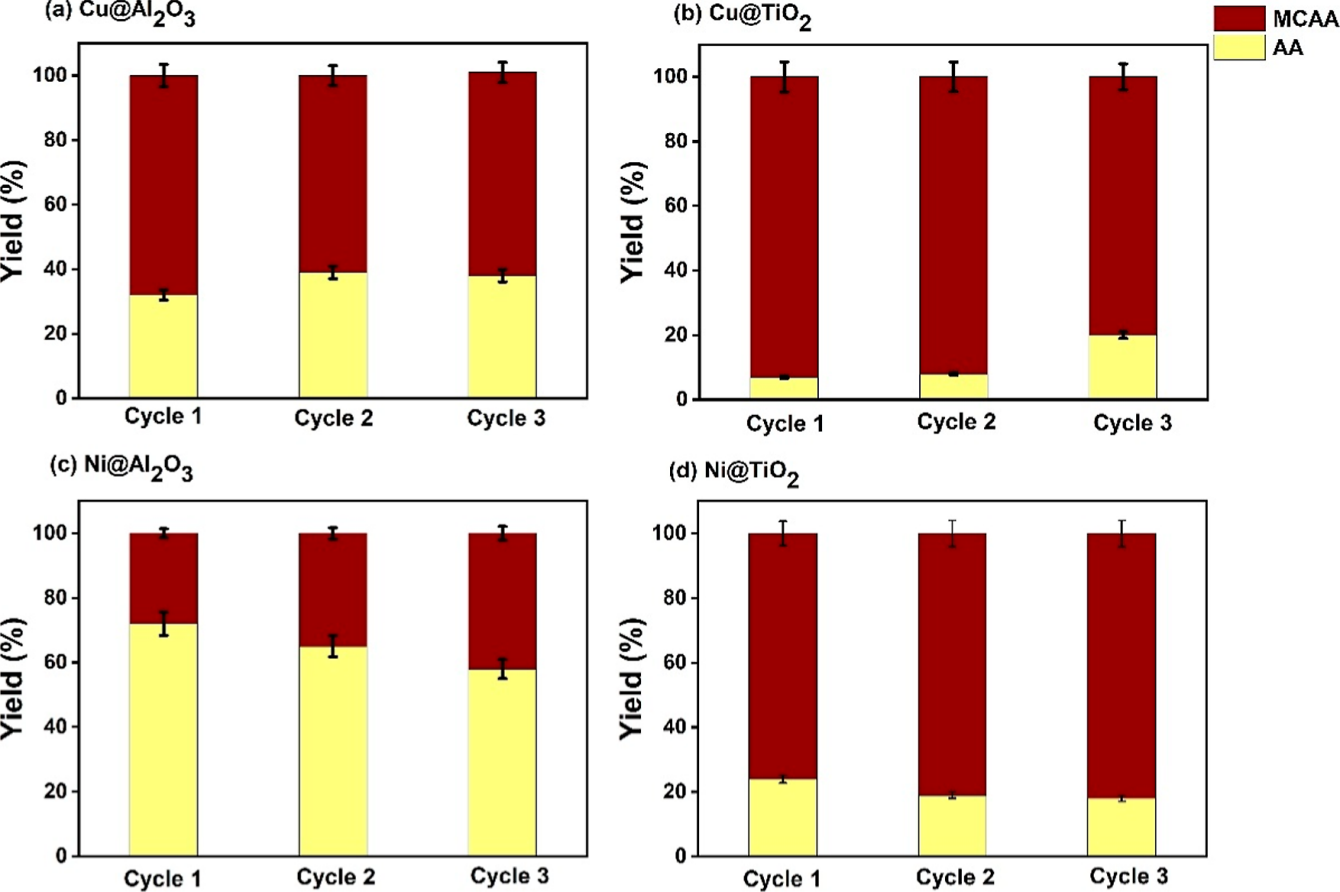
MCAA reduction reusability test results for 3 consecutive cycles of (a) 1% Cu­(II)@Al_2_O_3_, (b) 1% Cu­(II)@TiO_2_, (c) 1% Ni­(II)@Al_2_O_3_, and (d) 1% Ni­(II)@TiO_2_.

#### TCAA Reduction Reaction

Every catalyst employed reduces TCAA into MCAA and AA, leaving no DCAA or TCAA. Minor products involving FA and MA were also detected predominantly in the case of Ni­(II) encapsulated precatalysts, as was previously reported about the distinct behavior of Ni directing the formation of dimerized products during dehalogenation of trihalo-acetic acids.^16^ The results can be viewed from two perspectives: if one considers AA as the major product, the trend follows the following order: Cu­(II)@SiO_2_ > Ni­(II)@Al_2_O_3_ > Cu­(II)@Al_2_O_3_ > Ni­(II)@TiO_2_ ∼ Ni­(II)@SiO_2_ > Cu­(II)@TiO_2_, a trend that almost follows the case of MCAA reduction. But if one considers MCAA yields vs the yields of fully dehalogenated products (AA + FA/MA), the trend in the catalysts efficiency are Cu­(II)@SiO_2_ > Cu­(II)@Al_2_O_3_ > Cu­(II)@TiO_2_ in case of Cu and Ni­(II)@TiO_2_ > Ni­(II)@SiO_2_ ∼ Ni­(II)@Al_2_O_3_, in case of Ni (Figure 11), i.e., again the order of activities depends on the nature of the catalyst.

**Figure 11:**
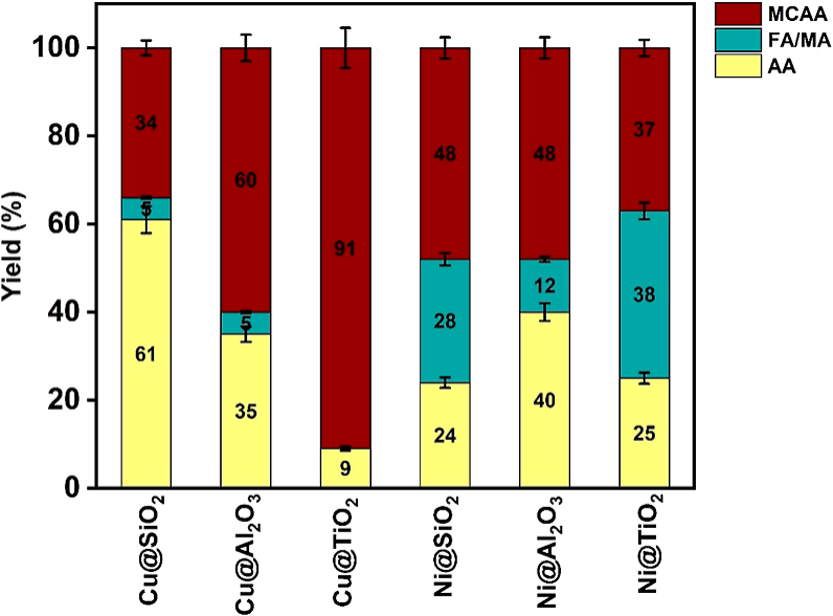
Product distribution in % of TCAA reduction. (Catalyst amount 0.5 g, [TCAA] = 0.05 M, [NaBH_4_] = 1.0 M, 0.57 g.)

The recyclability tests exhibit a pattern similar to that of MCAA reduction, with Ni-based catalysts showing a slight decrease in activity (Figure 12).

**Figure 12:**
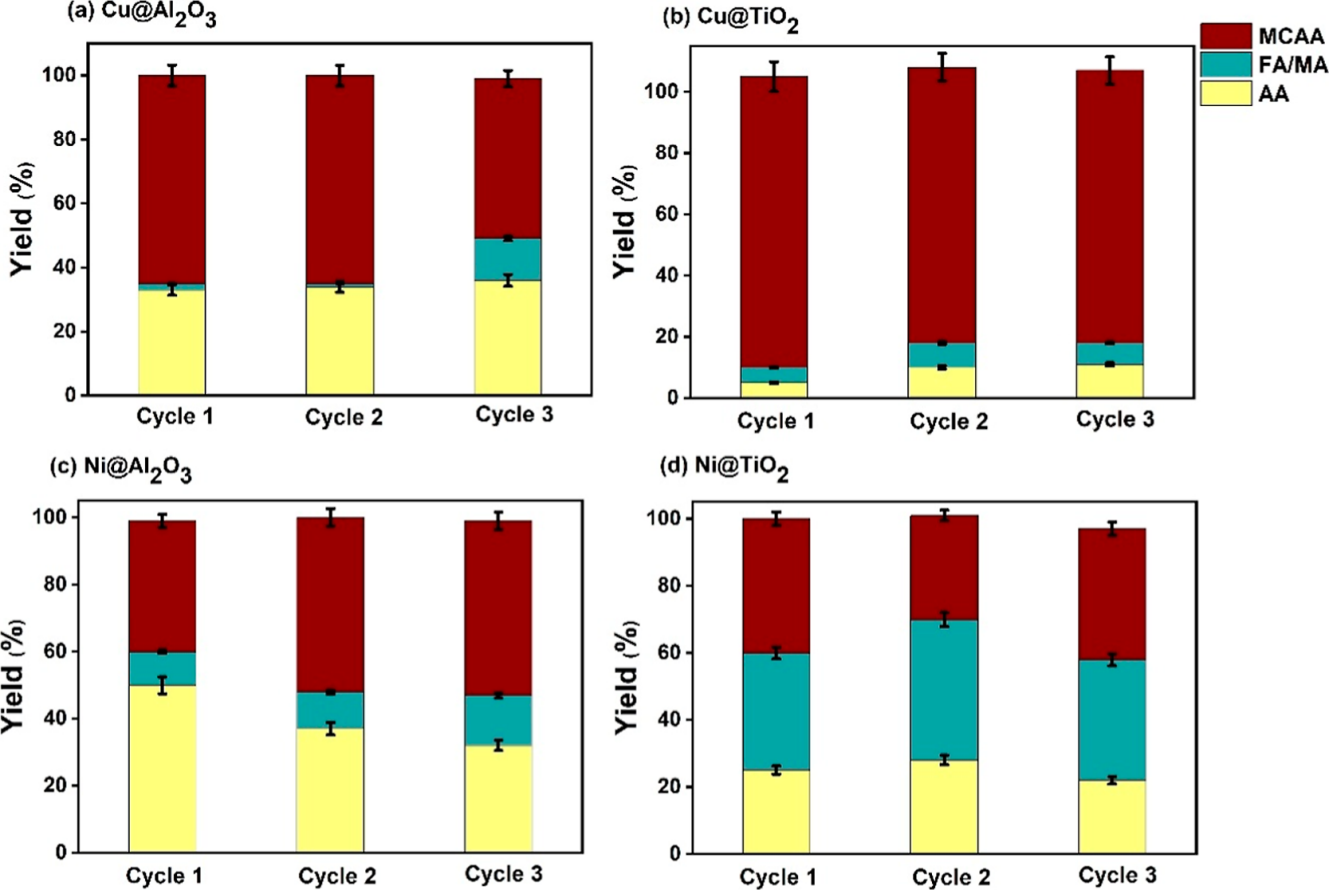
TCAA reduction reusability test results for 3 consecutive cycles of (a)­1% Cu­(II)@Al_2_O_3_ , (b) 1% Cu­(II)@TiO_2_1% , (c) 1% Ni­(II)@Al_2_O_3_, and (d) 1% Ni­(II)@TiO_2_.

#### TBAA Reduction Reaction

TBAA reduction reactions are more complex and different from the trend observed in dechlorination reactions. The efficiency in converting TBAA primarily to AA follows the order: Cu@Al_2_O_3_ > Cu@TiO_2_ > Cu@SiO_2_ > Ni@Al_2_O_3_ > Ni@SiO_2_ > Ni@TiO_2_. Cu, in this case, performed well in dehalogenating into AA on all three metal-oxide systems. Compared with Cu, the reduction of TBAA by Ni is not that efficient since the products still contain the brominated compounds DBAA, MBAA, and bromo-fumaric acid (BFA), Figure 13.

**Figure 13:**
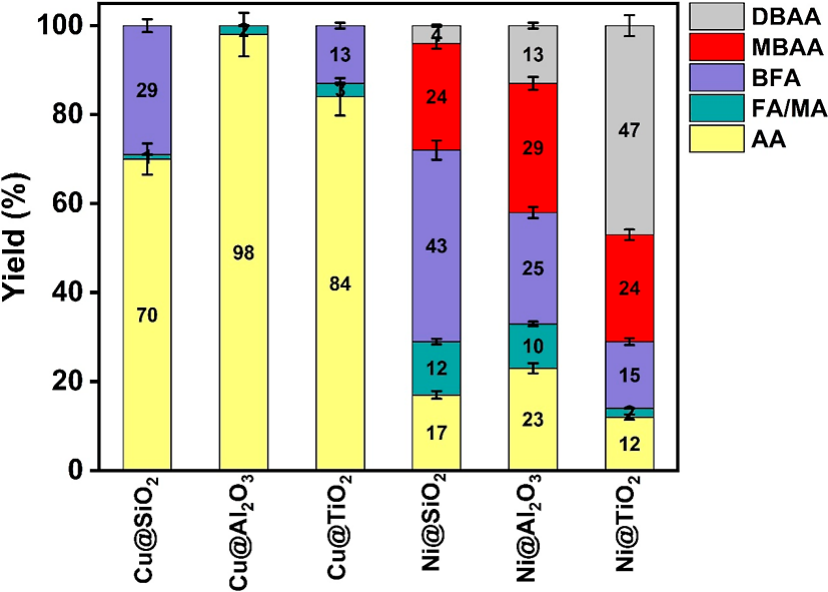
Product distribution in % of TBAA reduction. (Catalyst amount = 0.025 g, [TBAA] = 0.05 M, [NaBH_4_] = 0.3 M, 0.17 g.)

The formation of FA, MA, and BFA via the dehalogenations of TBAA/TCAA involves the dimerization of the bound radicals, CX_2_CO_2_^•–^/CXHCO_2_^•–^ (X = Cl/Br) on the surface of the M^0^ nanoparticles.^17^ This clearly competes with the heterolysis of the M–C bonds via reaction with water to yield MBAA and AA. The ratio [AA + MBA]/[FA + BFA] in the case of TBAA and [AA + MCA]/[FA/MA] in the case of TCAA reductions indicates which mechanism dominates over the other, Table 2. The dimerization of the radicals during TCAA reduction is more promoted by Ni in TiO_2_ > SiO_2_ > Al_2_O_3_ than by Cu catalysts. However, in the case of TBAA reduction, the SiO_2_ surface leads to more dimerization of the radicals than titania for both metals. The lower amount of MCAA remaining in the case of TCAA reduction than from MCAA reduction in the case of Ni@TiO_2_, Ni@SiO_2_, and Cu@SiO_2_ points out that on these catalysts, the heterolysis of the M–C bond in M^0^-CHClCO_2_^–^ is slower than the further dechlorination and/or the dimerization of the CHClCO_2_^–^ groups. These differences most likely arise from the difference in the chemical environment of the metal species. It is unequivocally established that strong communication occurs between the entrapped and adsorbed metal and the pore walls of the metal-oxide-based sol–gel material. Furthermore, the product distribution can also vary depending on the reaction conditions like metal loading, concentration of the substrate, BH_4_^–^, etc.^17^

**Table 2:** Ratio of [AA + MBA]/[FA + BFA] in the Dehalogenation of TBAA and [AA + MCA]/[FA/MA] in the Reduction of TCAA

	[AA + MCA]/[FA/MA] (TCAA reduction)	[AA + MBA]/[FA + BFA] (TBAA reduction)
Cu@SiO_2_	19	2.3
Cu@Al_2_O_3_	19	49
Cu@TiO_2_	-	5.3
Ni@SiO_2_	2.6	0.7
Ni@Al_2_O_3_	7.3	1.5
Ni@TiO_2_	1.6	2.1

The dehalogenation of MBAA was also studied: each of the catalysts was able to reduce it completely to AA (100%), making no point of comparison in this scenario.

Thus, the catalytic activity as well as the selectivity of the different catalytic systems employed on different substrates show dramatic differences between them. The reason and the contributing factors behind these phenomena can be understood only by delving into the realm of interfaces formed between the in situ-formed M^0^ and the encapsulating pore walls. The origin of enhancement of reactivity or selectivity by the SMSI usually comes from either an electronic effect, i.e., electron transfer between the metal and the oxide support, and/or a geometric effect, encapsulation of the metal nanocatalyst in the support.^55^ Briefly, bond formation and dissociation are facilitated by the metallic catalysts, while the charge transfer to the reactants and intermediates is correlated to the acidic or ionic properties of the metal oxide support.^56^ Such similar interactions can be the possibilities here as well, the only difference being that the encapsulated/adsorbed M^0^ is interacting with the walls of the pore based on SiO_2_/Al_2_O_3_/TiO_2_.

The interaction between silanol groups and adsorbed or entrapped metal species on the surface is already established for the silica-based sol–gel catalysts.^12,16–18,57,58^ Factors like adsorption energy, pH, and hydrophobicity of the pore walls contribute to these interactions.

The electronic properties of the metal and the hosting metal oxide matrix have a major effect on its catalytic properties, as charge transfer can occur between the two, altering the metal-reactant interaction. In the systems studied herein, this charge transfer is evident from the XPS data (Table S1) showing binding energy shifts happening to the Cu 2p_3_ and Ni 2p_3_ on different systems in the increasing order of SiO_2_ > Al_2_O_3_ > TiO_2_. This points out the more electron-donating behavior of TiO_2_ > Al_2_O_3_ > SiO_2_. This makes sense, given that TiO_2_ is an n-type semiconductor with excess charge, whereas the other two oxides are insulators, less able to donate electrons.^59^ It should be pointed out that the interactions between the matrix skeleton and the M^0^-NPs that catalyze the dehalogenation processes might differ considerably.

A look into the surface area shows that silica-based catalysts have a higher surface area than alumina and titania. Also, the more amorphous nature of silica may facilitate metal migration and thus more encapsulation and/or dispersion. Furthermore, this probably results in the formation of smaller M^0^-NPs, assuming that the M­(II) precatalysts are distributed homogeneously on the pore surfaces. From the XRF data, Table S2, the better encapsulation of Cu follows the order: Al_2_O_3_ > SiO_2_ > TiO_2_ and Ni: Al_2_O_3_ > TiO_2_ > SiO_2_. As we correlate this to their activity, it implies that the increased surface area and better encapsulation contribute to an increased catalytic performance for both Cu and Ni in silica and alumina compared to those in titania. This can also be explained in the light of pore size increment in the order of Al_2_O_3_ > SiO_2_ > TiO_2_ because a wide pore size facilitates the diffusion of bulk substrate molecules to the active centers, converting them into products.^60^

Not just these, the nature and behavior of the metals on each sol–gel material play a crucial role in altering their activities, as can be seen from their XRD data. The transition of γ-Al_2_O_3_ to its hydroxide form (Al­(OH)_3_) after a cycle of reaction in the case of Cu and not in the case of Ni and the structural modifications that happen to Ni after treatment with NaBH_4_ leads to the conclusion that different cations/metals are coordinated differently with each metal oxide matrix. Thus, the results point out that the different cations bind differently to the pore walls; for example, as Cu^2+^ and Ni^2+^ in alumina and as CuO and Ni­(TiO_3_) in titania. The more amorphous nature of silica and the crystallinity of Al_2_O_3_ and TiO_2_ can lead to the different reducibility of each metal on the surface.^61^ This can be seen from the XPS data, which show surface depletion of Cu^2+^ on Al_2_O_3_ and Ni^2+^ on both Al_2_O_3_ and TiO_2_ after a reaction cycle, resulting in a slight reduction in their activity in subsequent cycles.

The role and impact of the reactant molecules also must be considered. It has been shown that chlorinated organic compounds can be adsorbed and activated not only on metal surfaces but also on the encapsulating metal-oxide surface, while the role of the metal is to activate the borohydride and supply adsorbed hydrogen atoms to the substrates.^61,62^ One observes contradicting results for TBAA and TCAA reduction by the same metal on different metal-oxide systems. For example, for catalysis by Ni in TBAA reduction, more dimerization is observed in the order: SiO_2_ > Al_2_O_3_ > TiO_2_, while for TCAA, the order is TiO_2_ > SiO_2_ > Al_2_O_3_. In the present systems of dehalogenating HAAs using BH_4_^–^, many factors must be considered, e.g.:

a. The competitive adsorption of the substrate molecule and BH_4_^–^.
b. Hydrolysis mechanism of NaBH_4_.
c. Hydrogen evolution reaction (HER) and the overpotential required to enable HER.
d. H adsorption and diffusion.
e. Redox potential of M^0^.
f. The nature and charge of M^0^ nanoparticle.
g. Binding and diffusion of radicals on the M^0^ surface.
h. Activation energy of the dimerization of two −CX_2_CO_2_^–^/–CHXCO_2_^–^ neighboring groups.^63^

The HER process, though with Au^0^-NPs, is considerably faster for Au^0^-NPs@TiO_2_ than for Au^0^-NPs@SiO_2_.^12^ This clearly affects the processes studied herein. However, the effect of the sol–gel system on all of the other steps has to be studied.

The early studies on silica-based catalysts with different metals (Cu, Co, Fe, and Ni) have demonstrated the impact of these factors on product formation and distribution under different conditions of substrate and BH_4_^–^ concentrations.^17^ Detailed explanations regarding the mechanistic pathways followed in the dehalogenation reactions with the silica-based catalyst systems can be found in our earlier reports (Scheme S2).^16–18^ A concise schematic representation of the reduction mechanism is given (Scheme 2).

**Scheme 2:**
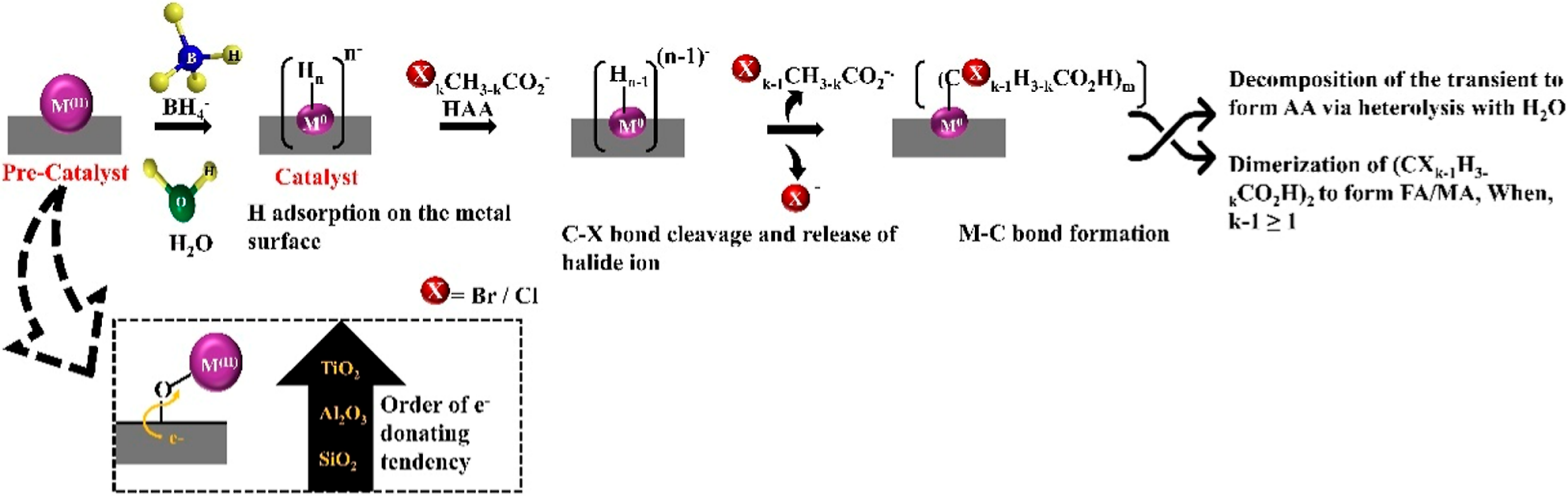
A Plausible Catalytic Reduction Mechanism for the Dehalogenation Reaction by the M^0^ Surface

The matrix probably affects all of the reaction steps discussed above and might differ for each step in the catalytic process. Furthermore, an increase in the pore surface area is expected to result in the formation of smaller M^0^-NPs, which affect their chemical properties. Therefore, to gain further mechanistic insights into the tuning of catalytic activity at the molecular level, one has to get a closer look at the reactive interfaces formed between the transition metal atoms/particles and the metal oxide surrounding it.

## Concluding Remarks

The effect of SiO_2_, Al_2_O_3_, and TiO_2_ on the M^0^-NPs catalyzed dehalogenation of HAAs by BH_4_^–^ was studied. The results indicate that the yields of the Cu catalyzed dechlorination of MCAA and TCAA decrease along the series SiO_2_ > Al_2_O_3_ > TiO_2_; however, for the analogous processes catalyzed by Ni^0^-NPs, the order of reactivities is Al_2_O_3_ > SiO_2_ > TiO_2_ for the reduction of MCAA and Al_2_O_3_ > TiO_2_ ∼ SiO_2_ for the reduction of TCAA. The yield of AA in the debromination of TBAA catalyzed by Cu^0^-NPs decreases along the series Al_2_O_3_ > TiO_2_ > SiO_2_, whereas for the analogous process catalyzed by Ni^0^-NPs, the order is Al_2_O_3_ > SiO_2_ > TiO_2_. If the desired product in the Ni^0^-NPs catalyzed debromination of TBAA is BFA, then the order of the product yield is SiO_2_ > Al_2_O_3_ > TiO_2_. The results point out that the surrounding chemical environment of the metal particles is considerably more complex than just electron transfer. Probably, each metal-oxide environment affects differently the sizes and surface properties of each M^0^-NP formed in situ within the pores. Thus, the results indicate that further studies are required to determine how the chemical environment around an active metal nanoparticle affects the different steps in these catalytic processes.

## Supplementary Material



## Notes

Cation exchanged Cu­(II)@SiO_2_ and its reduction to Cu(0)@SiO_2_ by BH_4_^–^, SEM/EDS images of the catalysts studied, N_2_ adsorption–desorption isotherms and BJH pore distribution plots of the catalysts studied, Raman spectra of the catalysts, full scan XPS spectra of the catalysts, XPS data of the catalysts, XRF data of the catalysts, ICP data, and plausible mechanistic pathway for the dehalogenation reaction on M^0^ surfaces (PDF)
