# Supplementary material for: The Sol–Gel Metal-Oxide Skeleton Affects the Catalytic Properties of In Situ Formed Metal Nanoparticles
Source: ACS Omega. 2026 Feb 17;11(8):13107–20. doi: 10.1021/acsomega.5c07438 (PMC12961463; doi:10.1021/acsomega.5c07438)
Supplement: Supplementary file 1 [file ao5c07438_si_001.pdf]

The Sol-Gel Metal-Oxide Skeleton Affects the Catalytic Properties of in-situ formed Metal Nanoparticles

Kavya Vidyadharan,[1] *Dan Meyerstein,*[1,2] *Ariela Burg,*[3] *Amir Mizrahi,*[4] *Jennifer Strunk,*[5] *Yael Albo*[6]*

[1]Chemical Sciences Department and The Radical Research Center, Ariel University, Ariel, 4070000, Israel.

[2]Chemistry Department, Ben-Gurion University, Beer-Sheva, 8410501, Israel.

[3] Chemical Engineering Department, Sami Shamoon College of Engineering, Beer-Sheva, 84100, Israel

[4]Nuclear Research Centre Negev, Beer-Sheva, 84190, Israel

[5]Industrielle Chemie und Heterogene Katalyse, Technische Universität München Lichtenbergstr. 4, 85748 Garching b. München, Germany.

[6]Chemical Engineering Department and The Radical Research Center, Ariel University, Ariel, 4070000, Israel.

Supporting Information

Scheme S1. The cation exchanged Cu(II)@SiO_2 and its reduction to Cu(0)@SiO_2 by BH_4^-.

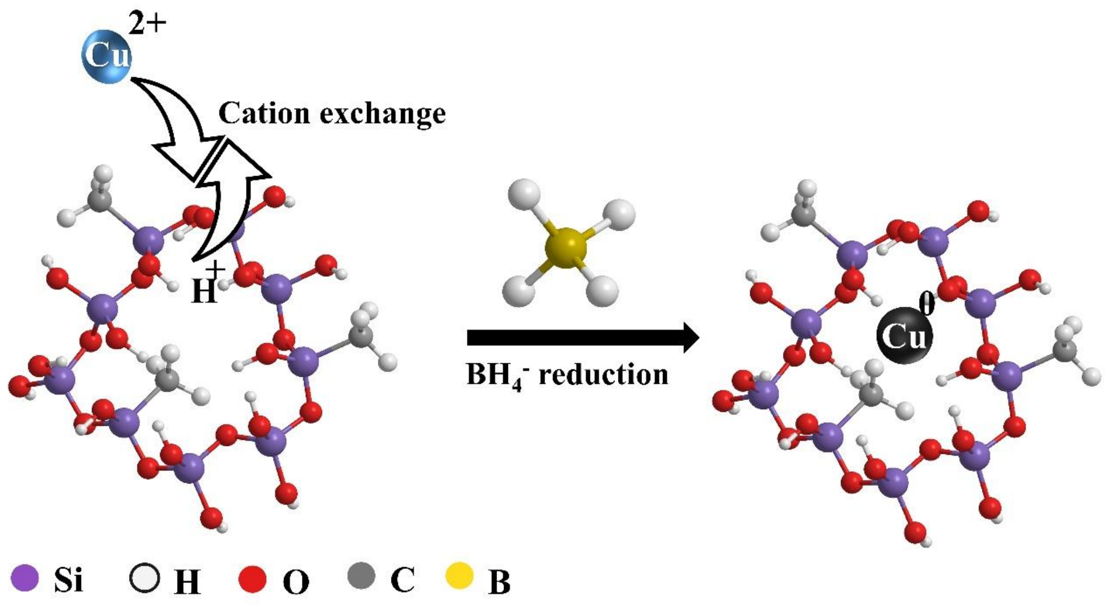

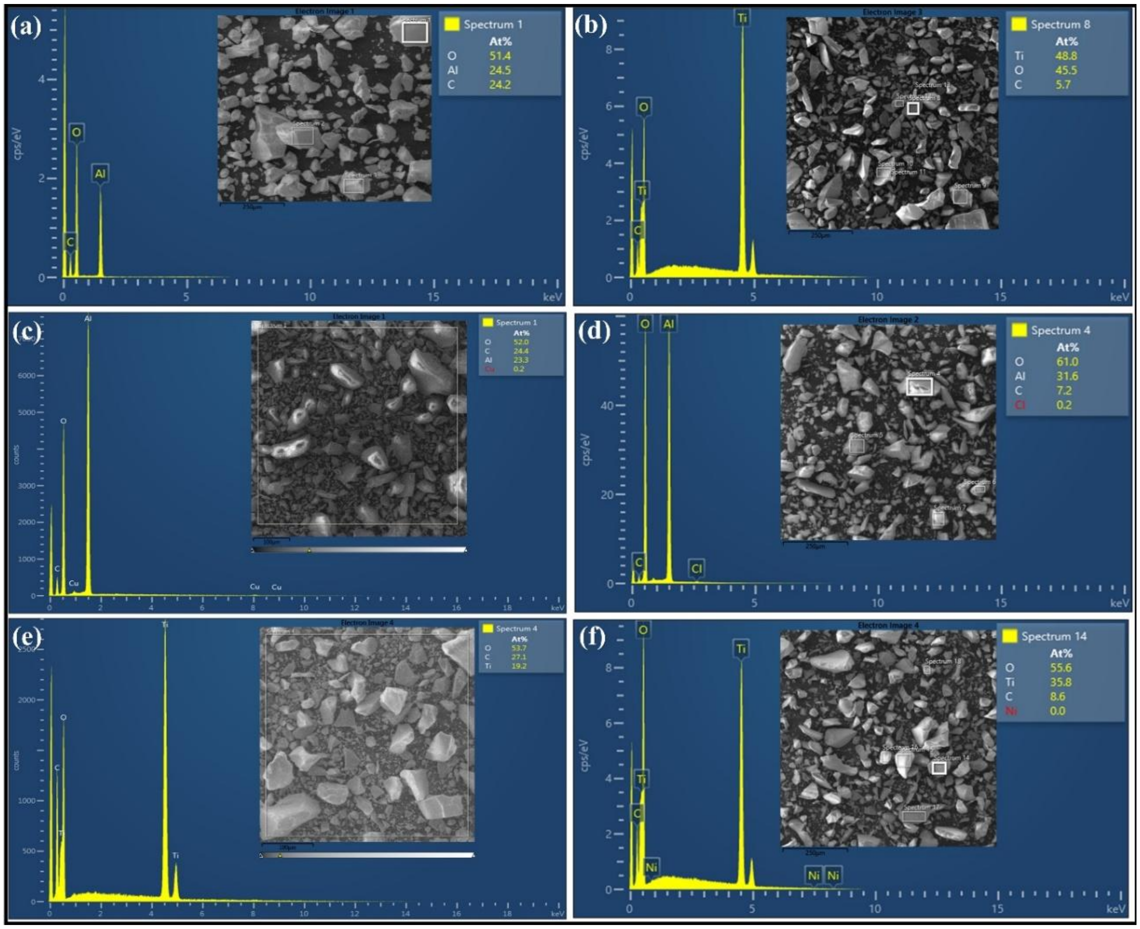

Figure S1. SEM/EDS images of (a) Blank Alumina (Al_2O_3) (b) Blank titania (TiO_2) (c) 1% Cu(II)@Al_2O_3 (d) 1% Ni(II)@Al_2O_3 (e) 1% Cu(II)@TiO_2 (f) 1% Ni(II)@TiO_2

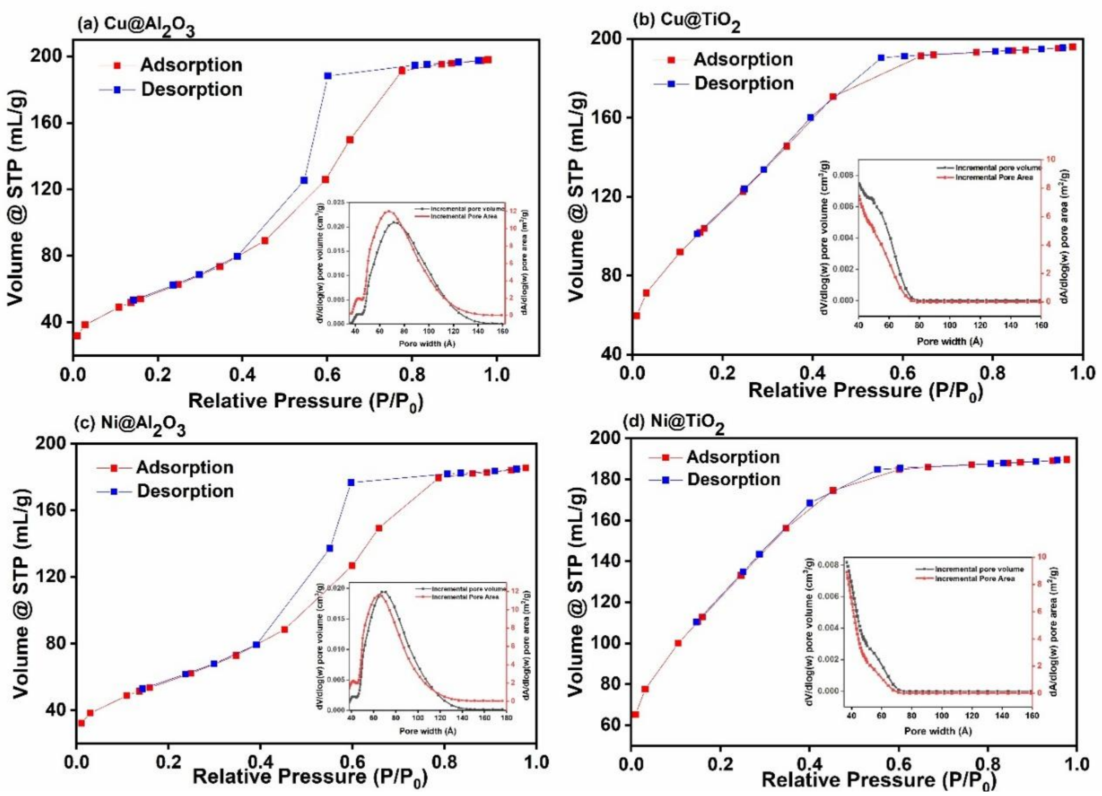

Figure S2. N2 adsorption-desorption isotherms of (a) 1% Cu(II)@Al2O3 (b) Cu(II)@TiO2 (c) 1% Ni(II)@Al2O3 (d) 1% (f) 1% Ni(II)@TiO2 . BJH pore-distribution curves are provided in the inset for each catalyst.

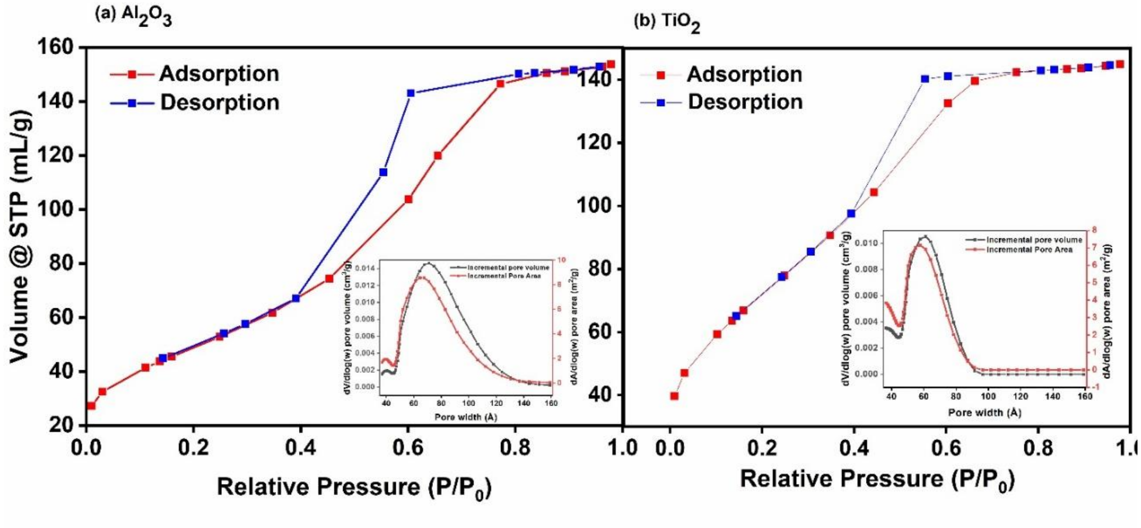

Figure S3. N2 adsorption-desorption isotherms of (a) Al_2O_3 (b) TiO_2. BJH pore-distribution curves are provided in the inset for each.

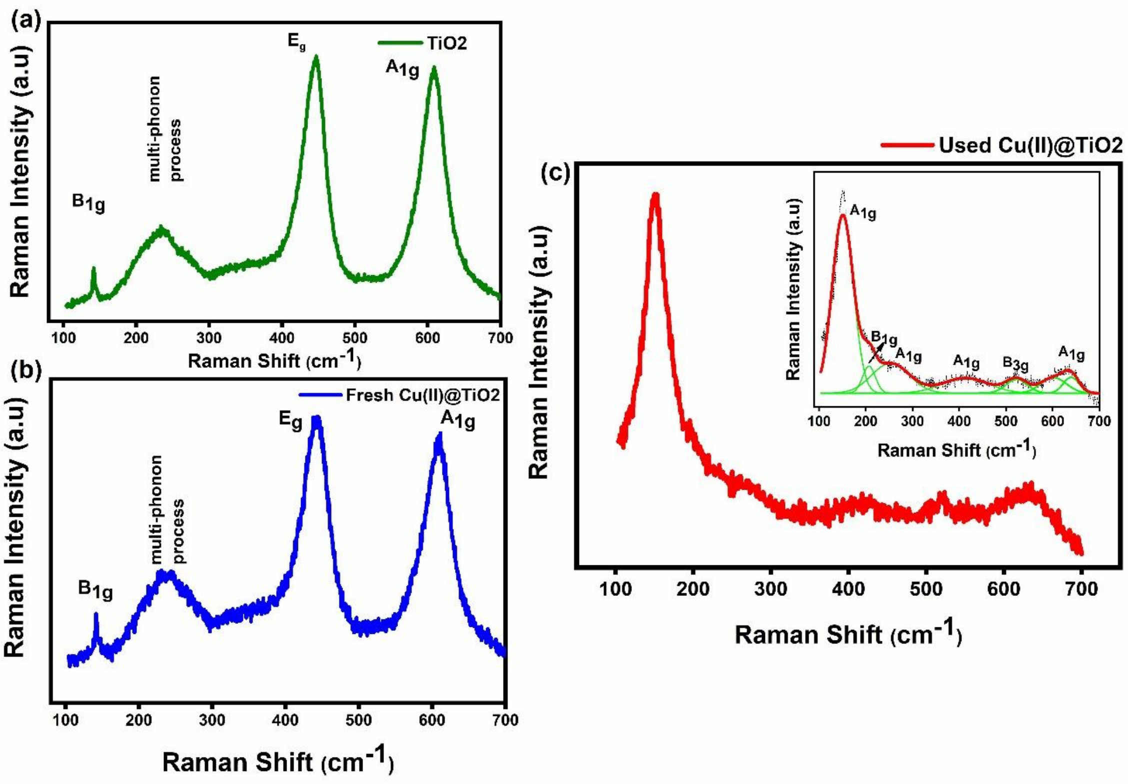

Figure S4. Raman spectra of the catalysts studied (a) Blank TiO_2 (rutile phase), (b) fresh Cu(II)@TiO_2 (rutile phase), (c) used Cu(II)@TiO_2 (brookite phase); Deconvoluted spectra of used Cu(II)@TiO_2 (inset)

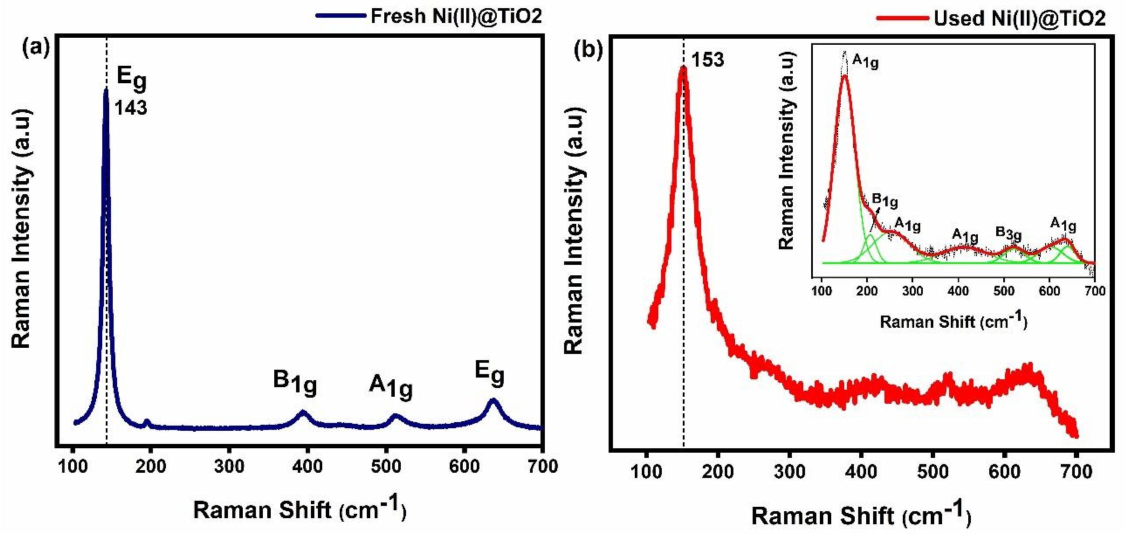

Figure S5. Raman spectra of the catalysts studied (a) fresh Ni(II)@TiO_2 (Anatase phase), (b) Used Ni(II)@TiO_2 (brookite phase); deconvoluted spectra of used Ni(II)@TiO_2 (inset).

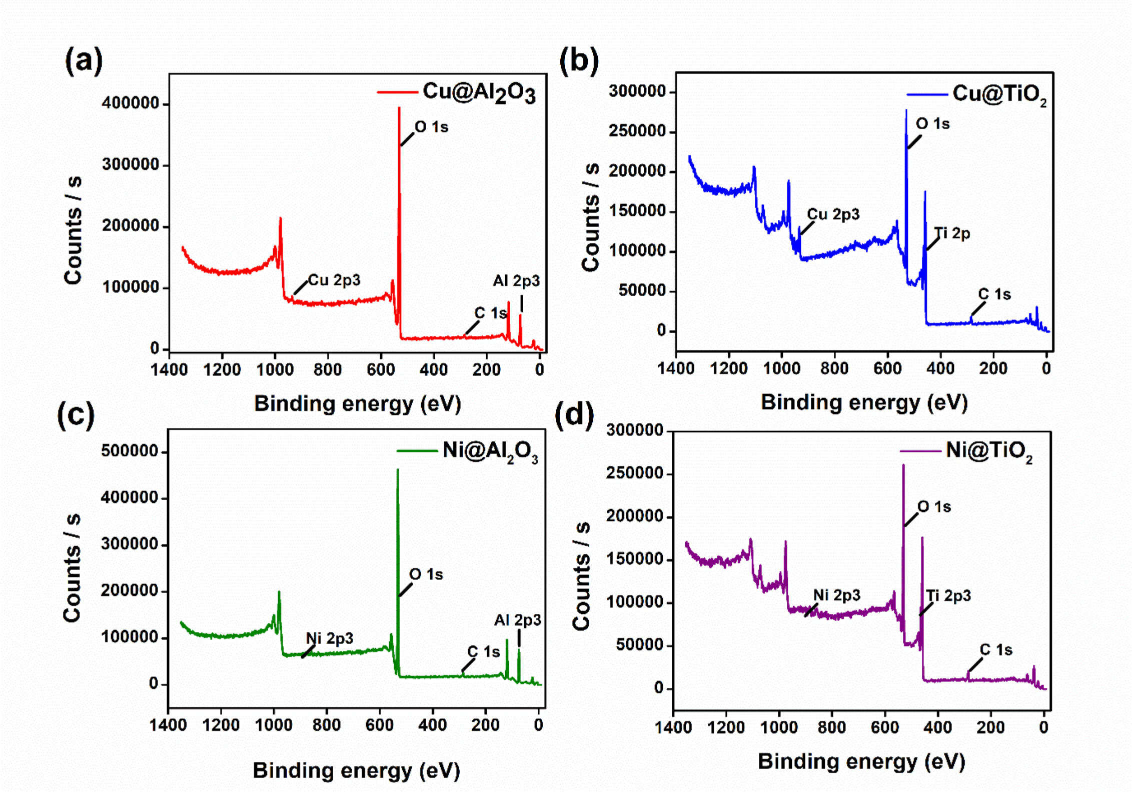

Figure S6. The full scan XPS spectra of (a) 1% Cu(II)@Al_2O_3 (b) 1% Cu(II)@TiO_2 (c) 1% Ni(II)@Al_2O_3 (d) 1% Ni(II)@TiO_2

Table S1. XPS data of the catalysts

Catalyst	element	Binding energy (eV)	XPS Atomic (%)
**1% Cu(II)@Al_2O_3**	Cu $2p_3$	933.82	0.5
	Al $2p_3$	73.43	35.5
	O1s	531.86	60.7
	C1s	285.01	3.3
**1% Cu(II)@TiO_2**	Cu $2p_3$	932.99	4.7
	Ti $2p_3$	458.18	28.7
	O1s	529.32	58.9

	C1s	284.74	7.8
**1% Cu(II)@SiO_2**	Cu $2p_3$	936.12	0.2
	Si $2p_3$	103.13	31.5
	O1s	535.3	57.7
	C1s	286.68	10.6
**1% Ni(II)@Al_2O_3**	Ni $2p_3$	857.28	0.4
	Al $2p_3$	74.48	35.8
	O1s	531.91	60
	C1s	284.86	3.8
**1% Ni(II)@TiO_2**	Ni $2p_3$	856.63	1.5
	Ti $2p_3$	459.02	29.2
	O1s	530.30	58.0
	C1s	285.71	11.2
**1% Ni(II)@SiO_2**	Ni $2p_3$*	-	-
	Si $2p_3$	103.18	30.4
	O1s	532.78	60.0
	C1s	284.86	9.6

*The Ni concentration on the surface was too low to be detected.

Table S2. XRF data of the catalysts

Catalyst	Element	mol.%
**1% Cu(II)@Al_2O_3**	Al	98.8
	Cu	1.2
**1% Cu(II)@TiO_2**	Ti	98.9
	Cu	1.1

**1% Cu(II)@SiO_2**	Si	99
	Cu	0.96
**1% Ni(II)@Al_2O_3**	Al	98.7
	Ni	1.3
**1% Ni(II)@TiO_2**	Ti	98.8
	Ni	1.2
**1% Ni(II)@SiO_2**	Si	99.8
	Ni	0.2

Table S3. ICP data of the silica-based catalysts

	**Expected concentration**	**Obtained concentration**
**Cu**	5 PPM	6.1
**(in 1% Cu@SiO2)**	10 PPM	11.6
**Ni**	5 PPM	3.4
**(in 1% Ni@SiO2)**	10 PPM	6.3

Scheme S2: A plausible mechanistic pathway for the de-halogenation reaction on M^0 surfaces

(Scheme reproduced with permission from American Chemical Society, copyright 2025. J. Phys. Chem. C 2025, 129, 7255−7262)

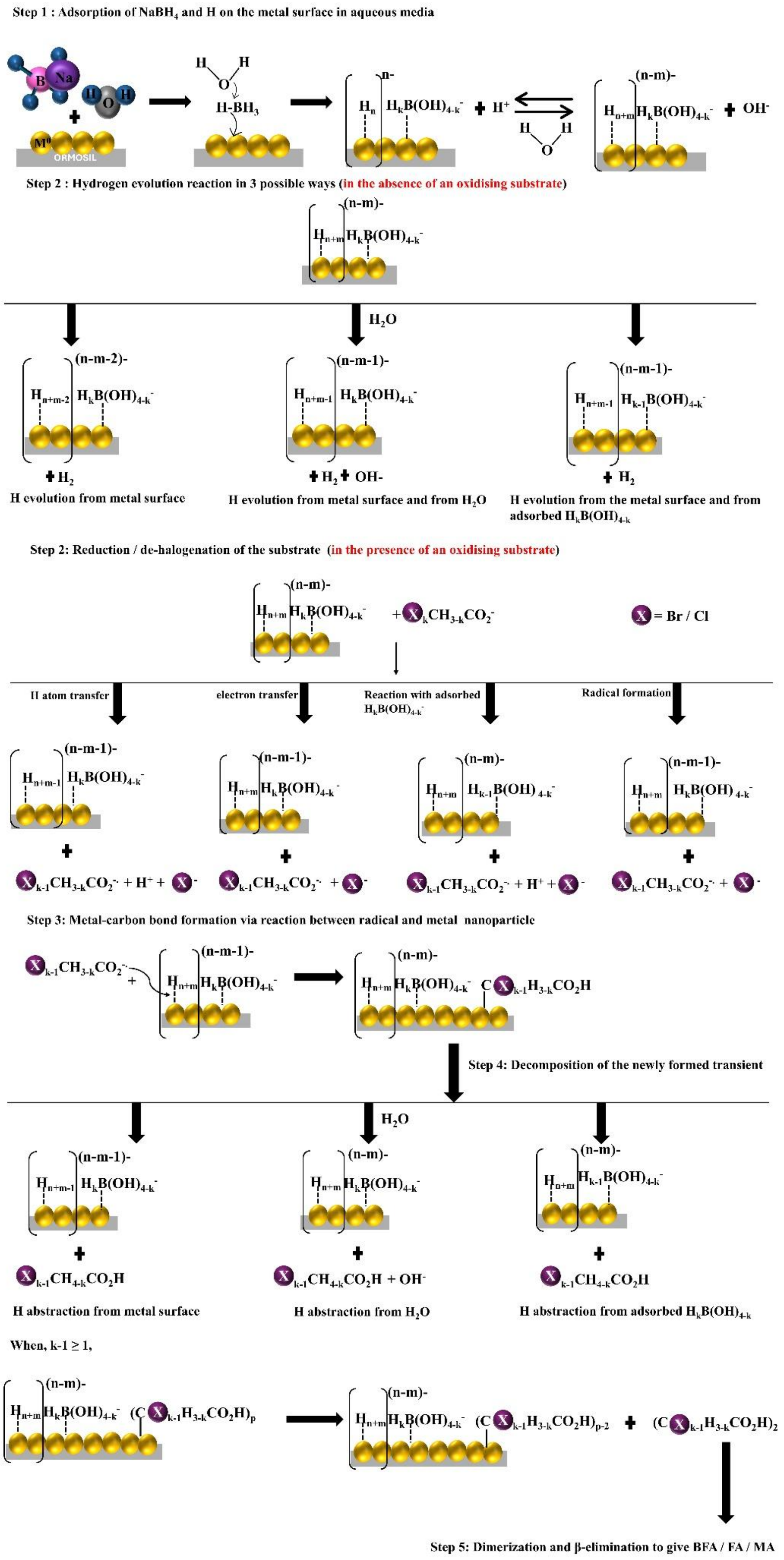
Step 1 : Adsorption of NaBH4 and H on the metal surface in aqueous media
ORMOSIL
Step 2 : Hydrogen evolution reaction in 3 possible ways (in the absence of an oxidising substrate)
H evolution from metal surface
H evolution from metal surface and from H2O
H evolution from the metal surface and from adsorbed HkB(OH)4-k
Step 2: Reduction / de-halogenation of the substrate (in the presence of an oxidising substrate)
X = Br / Cl
H atom transfer
electron transfer
Reaction with adsorbed HkB(OH)4-k-
Radical formation
Step 3: Metal-carbon bond formation via reaction between radical and metal nanoparticle
Step 4: Decomposition of the newly formed transient
H abstraction from metal surface
H abstraction from H2O
H abstraction from adsorbed HkB(OH)4-k
When, k-1 ≥ 1,
Step 5: Dimerization and β-elimination to give BFA / FA / MA
